# Supplementary material for: Functional connectivity patterns of the Giant Toad Rhinella horribilis in anthropogenically modified landscapes
Source: PLoS One. 2025 Oct 15;20(10):e0319111. doi: 10.1371/journal.pone.0319111 (PMC12527146; doi:10.1371/journal.pone.0319111)
Supplement: S1 File — (PDF) [file pone.0319111.s001.pdf]

Functional connectivity patterns of the giant toad *Rhinella horribilis* in anthropogenically modified landscapes

Gerardo J. Soria-Ortiz, Leticia M. Ochoa-Ochoa, Juan P. Jaramillo-Correa, Íñigo Martínez-Solano, Ella Vázquez-Domínguez

Supplementary material

Supplementary figures: S1 Fig – S6 Fig

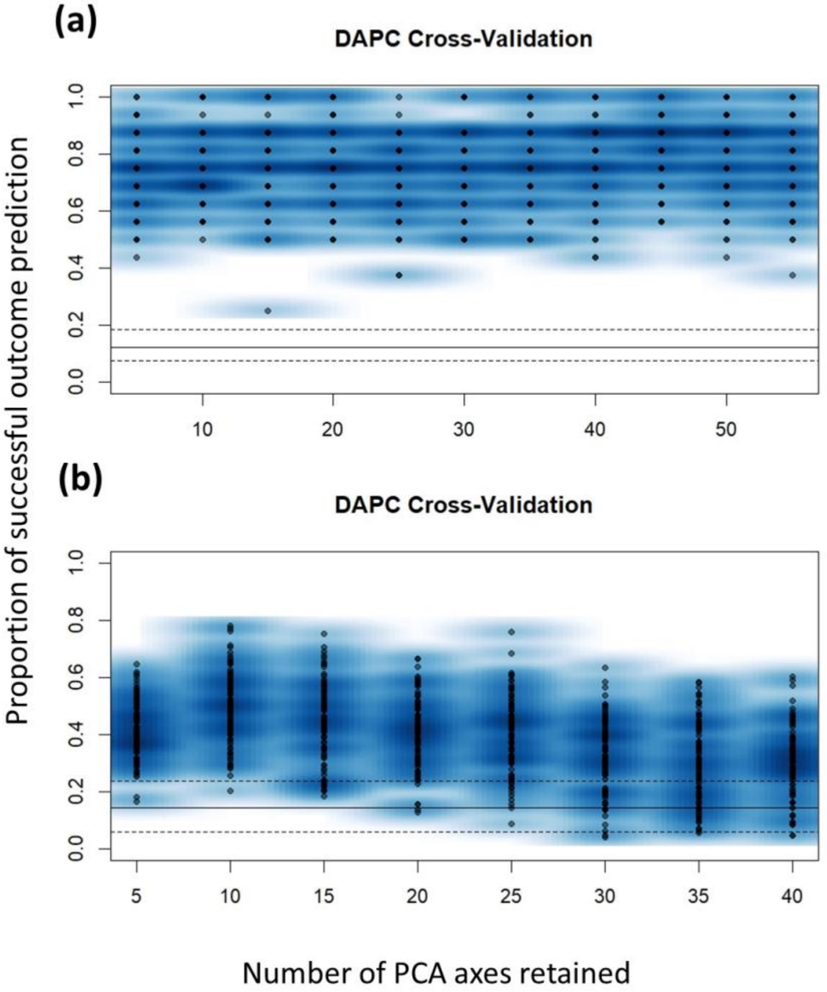

S1 Fig. DAPC cross-validation. The “x” axis shows the number of PCA axes retained for the DAPC and the “y” axis depicts the proportion of successful predictions. (**a**) Landscape 1 (P1O) and (**b)** landscape 2 (P2O).

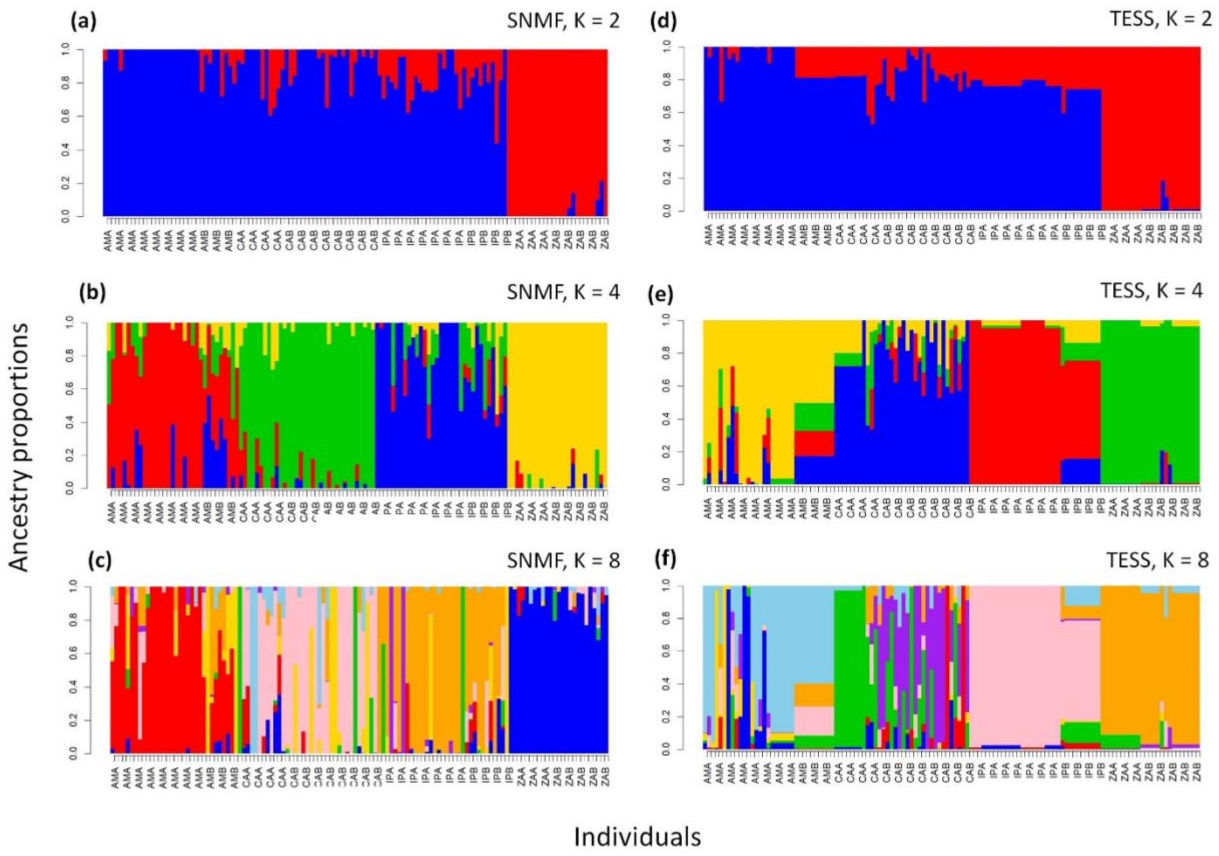

S2 Fig. Genetic differentiation of *Rhinella horribilis* in landscape 1 (P1O). Different structure plots performed with SNMF (**a-c**) and TESS (**d-f**) methods. Ancestry proportion results for *K*=2, 4 and 8.

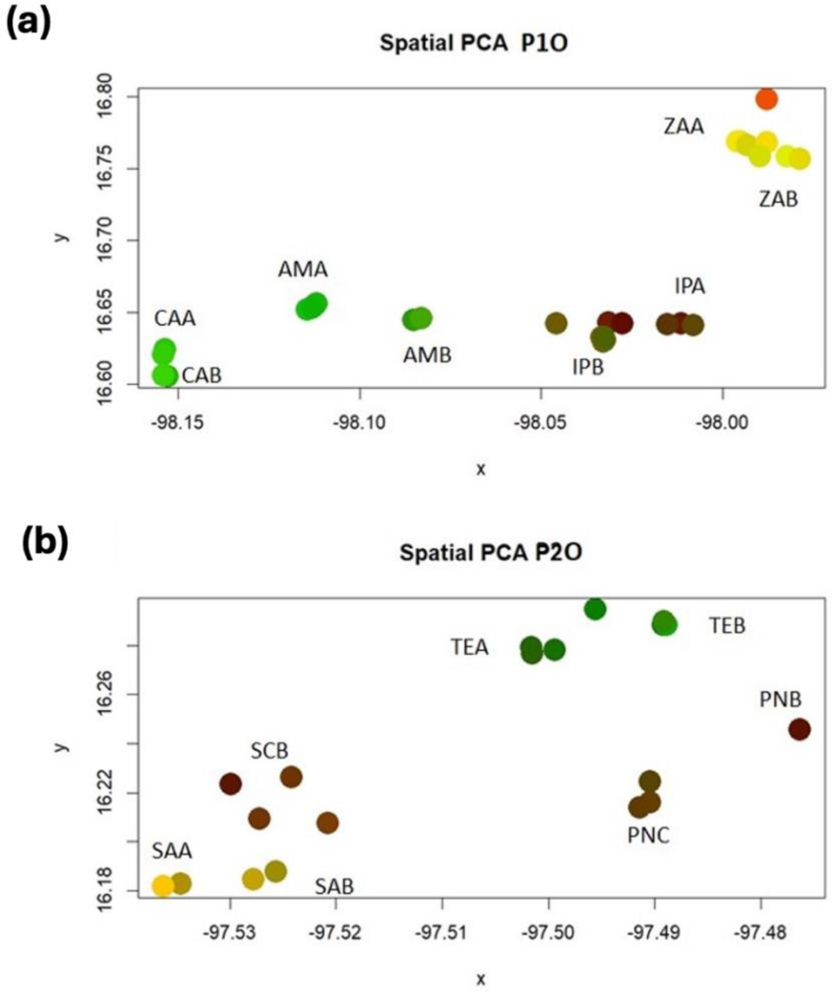

S3 Fig. Spatial principal component analyses (sPCA) for both landscapes. (**a**) Landscape 1 (P1O); (b) landscape 2 (P2O). Scale color indicates individuals that are closely related in the multivariate space.

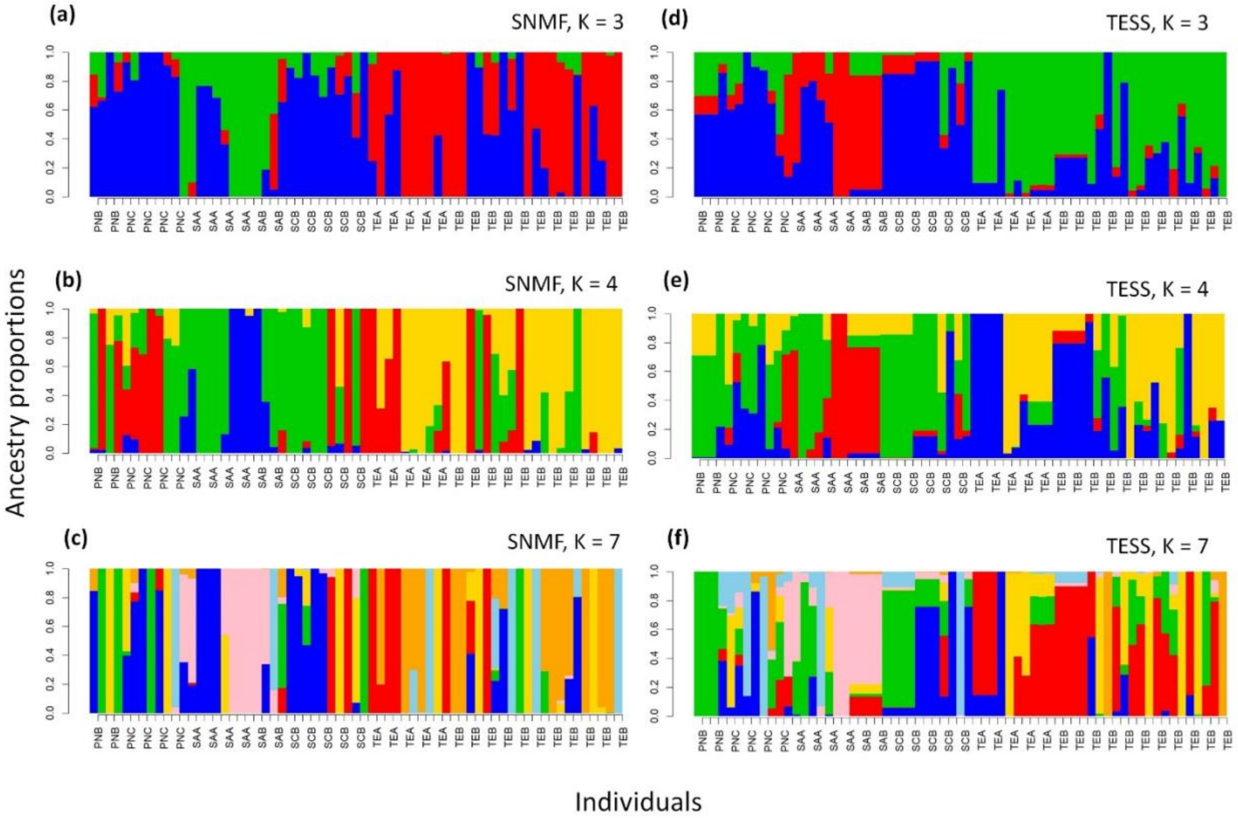

S4 Fig. Genetic differentiation of *Rhinella horribilis* in landscape 2 (P2O). Different structure plots performed for with SNMF (**a-c**) and TESS (**d-f**) methods. Ancestry proportion results for *K*=3, 4 and 7.

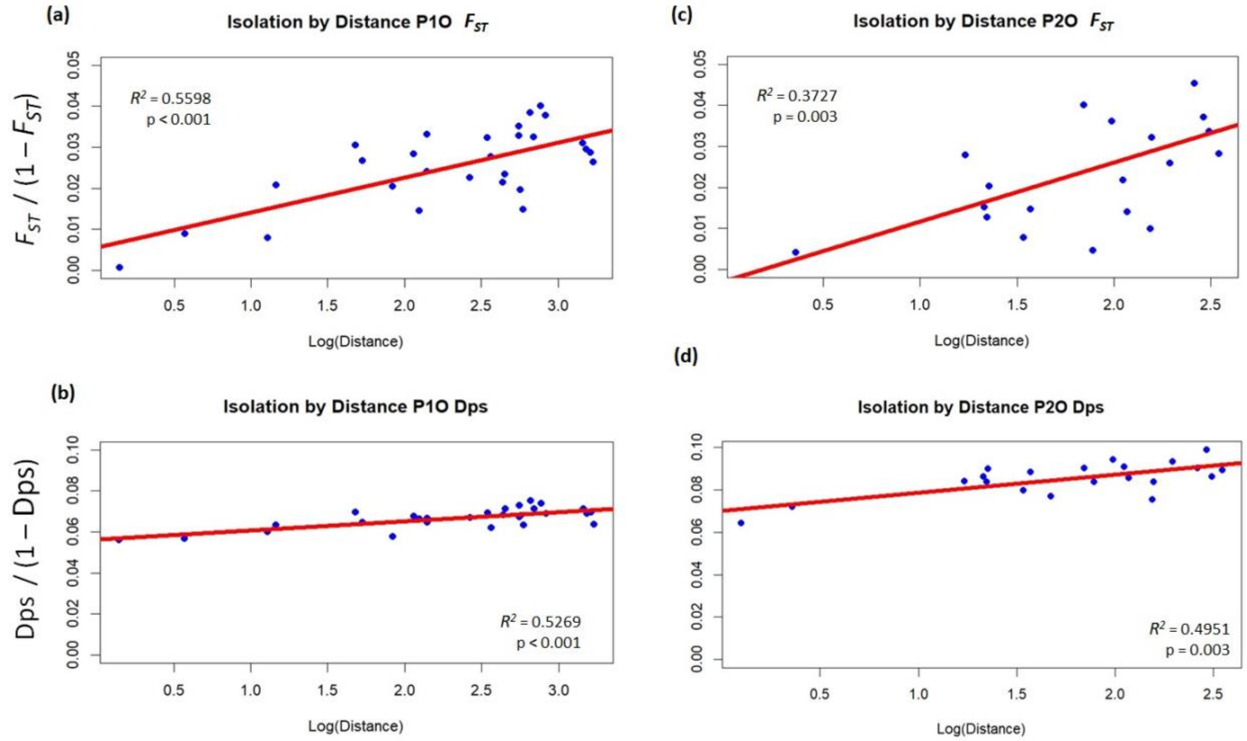

S5 Fig. Isolation by distance plots. Log of geographic Euclidian distance (km) on the "x" axis and genetic distance (F_{ST} and *Dps*) on the "y" axis, for (**a-b**) landscape 1 (P1O) and (**c-d**) landscape 2 (P2O).

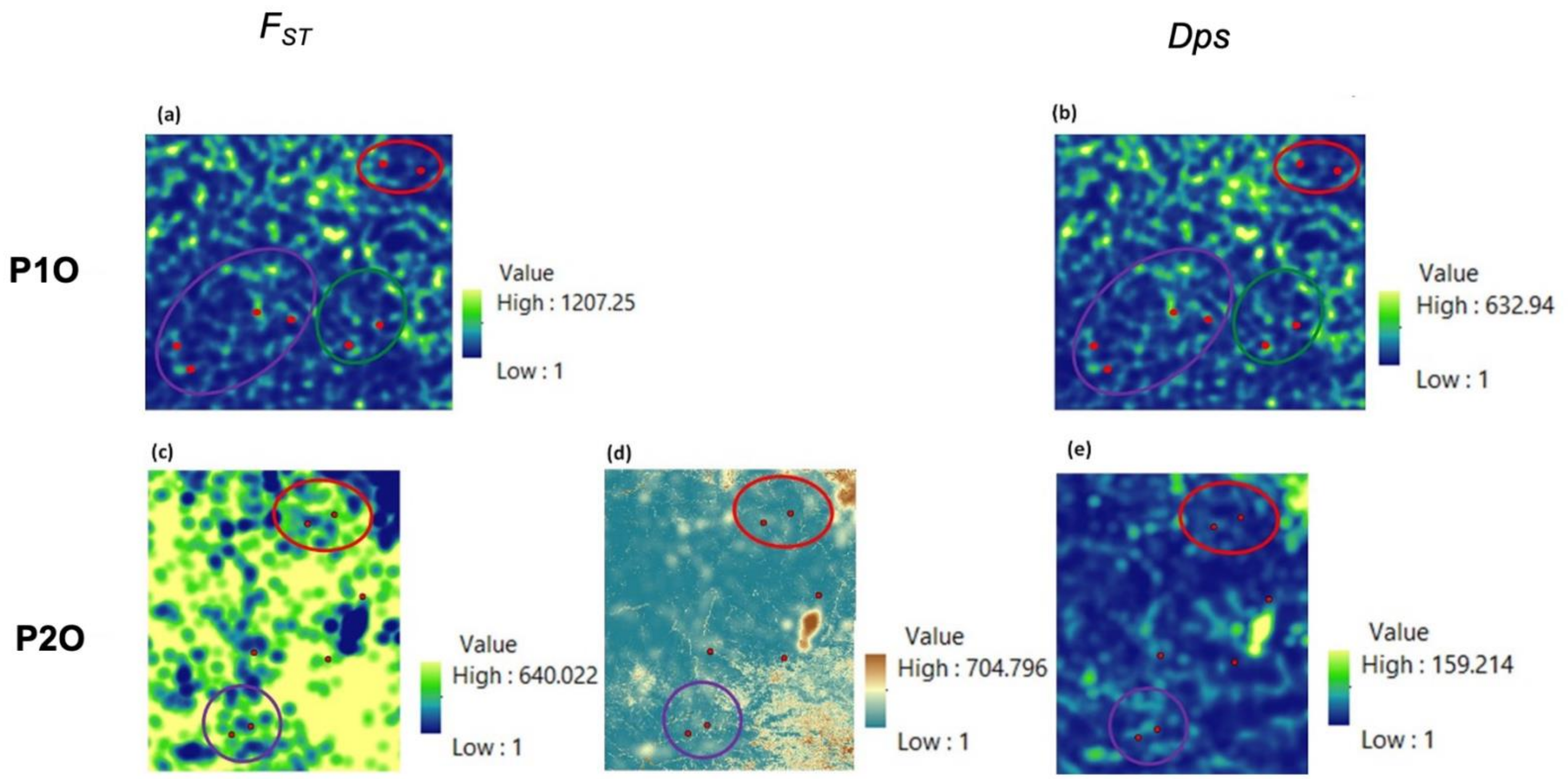

S6 Fig. Optimized resistance surfaces of the best supported multivariate models for *Rhinella horribilis* in Oaxaca, southern Mexico. Landscape P1O (upper images) and P2O (lower images), based F_{ST} (left; a,c,d) and *Dps* (right; b,e) (see Table 3). (**a**), (**b**), (**c**) and (**e**) Aquatic model (Temporary water bodies + Temporary streams); (**d**) Structural model (Temporary water bodies + Temporary streams + NDVI). Color scales in each inset depict resistance values. Dots indicate sampling sites, and red, green and purple ovals depict the genetic groups identified.
