# Supplementary material for: Functional connectivity patterns of the Giant Toad Rhinella horribilis in anthropogenically modified landscapes
Source: PLoS One. 2025 Oct 15;20(10):e0319111. doi: 10.1371/journal.pone.0319111 (PMC12527146; doi:10.1371/journal.pone.0319111)
Supplement: S2 File — (PDF) [file pone.0319111.s002.pdf]

Functional connectivity patterns of the giant toad *Rhinella horribilis* in anthropogenically modified landscapes

Gerardo J. Soria-Ortiz, Leticia M. Ochoa-Ochoa, Juan P. Jaramillo-Correa, Íñigo Martínez-Solano, Ella Vázquez-Domínguez

Supplementary material

Supplementary Tables S1-S7

Table S1. Class and landscape metrics that describe landscapes P1O and P2O. Contiguity index (CONTAG) indicates how disaggregated or aggregated are the patches of all land use classes within each landscape. Lower values indicate disaggregated patches (greater fragmentation); higher values indicate more aggregated patches (less fragmentation). The percentage of area (PLAND) denotes the percentage of each land class in the landscape, where low and high values indicate less and greater proportion of a land class. Vegetation cover encompasses dense and secondary forest, while Modified land includes urban area, bare soil and crops and pastures. Large patch index (LPI) shows the percentage of area of the largest patch of each land class, where high and small values are associated with large and small patches, respectively.

**P1O**				
Contiguity index (CONTAG) = 56.2				
Class	Percentage of area (PLAND)		Percentage of area (PLAND)	Large patch index (LPI)
Urban area	1.04			0.053
Dense forest	3.18	Vegetation cover	47.88	0.13
Secondary forest	44.7			11.55
Water bodies	0.41			0.06
Bare soil	17.45	Modified land	50.64	0.19
Crops and pastures	33.19			0.21
**P2O**				
Contiguity index (CONTAG) = 50.60				
Class	Percentage of area (PLAND)		Percentage of area (PLAND)	Large patch index (LPI)
Urban area	2.11			0.39
Dense forest	5.91	Vegetation cover	53.73	2.24
Secondary forest	47.82			18.15
Water bodies	0.58			0.16
Bare soil	5.32	Modified land	43.55	0.17
Crops and pastures	38.23			27.92

Table S2. Genetic diversity for *Rhinella horribilis* in landscape 1 (P1O) and landscape 2 (P2O) from Oaxaca, southern Mexico. Number of individuals (N) observed (H_o) and expected (H_e) heterozygosity and inbreeding (F_{IS}) values are indicated per sampling locality in each landscape. ± values: standard deviation

Landscape	Sampling site	Code	N	H_o	H_e	F_{IS}
**P1O**	Santa María Zacatepec	ZAA	23	0.111	0.120	0.078
		ZAB	10	0.111	0.117	0.067
	San Pedro Amuzgos	AMA	11	0.109	0.119	0.089
		AMB	23	0.110	0.116	0.053
	Santa María Ipalapa	IPA	23	0.108	0.123	0.113
		IPB	10	0.112	0.120	0.043
	San Juan Cacahuatepec	CAA	10	0.105	0.113	0.071
		CAB	15	0.110	0.120	0.074
**Total**			125	0.109 ±0.0022	0.118 ±0.0030	0.074 ±0.0214
**P2O**	Santa Ana Tututepec	SAA	8	0.143	0.151	0.098
		SAB	4	0.135	0.146	0.085
	Santa Cruz Tututepec	SCB	11	0.139	0.153	0.115
	Santa Cruz Tepenixtlahuaca	TEA	10	0.145	0.155	0.076
		TEB	21	0.144	0.162	0.128
	Peñas Negras	PNB	3	0.136	0.151	0.053
		PNC	8	0.137	0.155	0.109
**Total**			65	0.139 ±0.0040	0.153 ±0.0049	0.094 ±0.0255

Table S3. Pearson correlation results of the environmental surfaces tested in the functional connectivity models. The correlation test was performed independently for each landscape. Correlated variables (>±0.8) are in bold.

**P1O**										
	BSI_P1O	NDMI_P1O	NDVI_P1O	RH_P1O	TA_P1O	SR_P1O	EVA_P1O	ELV_P1O	TS_P1O	TWB_P1O
BSI_P1O	1									
NDMI_P1O	**-0.990**	1								
NDVI_P1O	**-0.892**	**0.847**	1							
RH_P1O	0.006	-0.003	-0.012	1						
TA_P1O	0.036	-0.030	-0.058	-0.434	1					
SR_P1O	-0.076	0.079	0.083	-0.450	0.0739	1				
EVA_P1O	0.081	-0.072	-0.072	-0.149	0.4201	0.3227	1			
ELV_P1O	-0.002	0.000	0.000	0.127	-0.1071	-0.1258	-0.0857	1		
TS_P1O	0.012	-0.008	0.003	-0.120	-0.0641	0.2586	0.1668	0.1207	1	
TWB_P1O	0.091	-0.092	-0.056	0.088	-0.2893	-0.0175	-0.3646	0.1101	0.113	1

**P2O**										
	BSI_P2O	NDMI_P2O	NDVI_P2O	TWB_P2O	TS_P2O	EVA_P2O	SR_P2O	ELV_P2O	RH_P2O	TA_P2O
BSI_P2O	1									
NDMI_P2O	**-0.994**	1								
NDVI_P2O	**-0.924**	**0.902**	1							
TWB_P2O	0.028	-0.019	-0.006	1						
TS_P2O	0.200	-0.199	-0.170	-0.074	1					
EVA_P2O	0.700	-0.696	-0.606	-0.077	0.316	1				
SR_P2O	0.609	-0.602	-0.507	0.002	0.212	**0.920**	1			
ELV_P2O	-0.698	0.695	0.609	0.107	-0.394	**-0.956**	**-0.876**	1		
RH_P2O	-0.535	0.525	0.441	-0.116	-0.282	-0.615	-0.570	0.601	1	
TA_P2O	0.366	-0.351	-0.270	-0.038	0.256	0.601	0.582	-0.530	-0.735	1

Table S4. Landscape variables used for resistance (connectivity) models for *Rhinella horribilis* in two landscapes in Oaxaca, southern México. Variable name, code and surface modeling, its source and original resolution are indicated, as well as Predicted positive (+) and negative (-) association. The corresponding biological justification of the effect of environmental variables on functional connectivity is included.

Variable	Code	Surface modeling	Source	Resolution		Biological justification
**Distance**	D	Euclidian distance (in meters) between populations			-	Distance between populations is a limiting factor in the connectivity of anuran populations (Murphy et al., 2010; Homola et al., 2019a; Chan & Brown, 2020)
**Temporal water bodies**	TWB	Kernel density of water bodies	Sentinel A2 image, infrared vision (Campton et al., 2007)	10 m	+	In areas with scarce vegetation, there is a greater likelihood for the formation of water bodies, which in modified environments are essential.<br>Areas with more water bodies will positively influence anuran connectivity (Campton et al., 2007)
**Temporal streams**	TS	Kernel density of temporary streams	INEGI	10 m	+	Temporary streams are associated with areas with land use change for agriculture.<br>Areas with more water bodies will positively influence anuran connectivity (Campton et al., 2007)
**Elevation**	ELEV	Mexican elevational continuum surfaces	INEGI	15 m	-	Elevation regulates connectivity, and reproductive and behavioral processes (Giordano et al., 2007; Murphy et al., 2010; Medina et al., 2021)
**Vegetation**	NDVI	Normalized vegetation index	Image Sentinel A2	10 m	-	Land use changes due to anthropogenic modification, changes in vegetation and decreasing tree cover.<br>*Rhinella horribilis* can occupy areas with low vegetation cover (Cortés-Suárez, 2017).
**Soil humidity**	NDMI	Normalized humidity index	Image Sentinel A2	10 m	+	In areas with little tree cover soils lose moisture at high rates. Moist soils determine the distribution and abundance of anurans; key for water conservation of anurans; *Rhinella horribilis* occurs in humid soils during the dry season (Cohen & Alford, 1996; Aryal et al., 2020)
**Impervious surface**	BSI	Bare soil index	Image Sentinel A2	10 m	-	The more anthropogenic modification of natural areas the greater extension of impervious surfaces<br>Abundance of *R. horribilis* is low or null in urbanized areas (Ramírez-Arce et al., 2022)

**Ambient temperature**	AT	Interpolated temperature surface	measured *in situ*		+	Areas with diminished tree cover combined with impervious surfaces are expected to experience increased ambient temperature.<br>*R. horribilis* tolerates high ambient temperature thresholds. It has been registered in 26°C sites (Cortés-Suárez, 2017).
**Relative humidity**	RH	Interpolated relative humidity surface	measured *in situ*	10 m	+	High humidity is associated with high vegetation cover and little modified areas.<br>Relative humidity is a limiting factor for *R. horribilis*, prefer humidities >80% (Cortés-Suárez, 2017).
**Solar radiation**	SR	5 years of solar radiation data	WorldClim	1 km	+	Higher incidence of solar radiation in areas with diminished vegetation cover due to anthropogenic modification.<br>Reports of coloration change in individuals/eggs exposed to UV rays. DNA repair in amphibians (Blaustein & Belden, 2003; Rollins et al., 2015)
**Evapotran spiration**	EVA	10 years of evapotranspiration data	WorldClim	1 km	-	Higher evapotranspiration in areas with diminished vegetation cover and more modified landscapes.<br>Anurans permeable skin makes them intolerant to water loss (Huang et al., 2016)
**Land use**	LU	Five classes of land use	Image Sentinel A2	10 m		Different land uses due to anthropogenic modification can limit, or sometimes facilitate, anuran connectivity (Covarrubias et al., 2021)

References

Aryal, P. C., Aryal, C., Neupane, S., Sharma, B., Dhamala, M. K., Khadka, D., ... & Neupane, D. (2020). Soil moisture & roads influence the occurrence of frogs in Kathmandu Valley, Nepal. *Global Ecology and Conservation*, 23, e01197.

Blaustein, A. R., & Belden, L. K. (2003). Amphibian defenses against ultraviolet-B radiation. *Evolution & Development*, 5(1), 89-97.

Covarrubias, S., González, C., Gutiérrez-Rodríguez, C. (2021). Effects of natural and anthropogenic features on functional connectivity of anurans: a review of landscape genetics studies in temperate, subtropical and tropical species. *Journal of Zoology*, 313(3), 159-171.

Campton, B. W., McGarigal, K., Cushman, S. A., & Gamble, L. R. (2007). A resistant-kernel model of connectivity for amphibians that breed in vernal pools. *Conservation Biology*, 21(3), 788-799.

Chan, K. O., & Brown, R. M. (2020). Elucidating the drivers of genetic differentiation in Malaysian torrent frogs (Anura: Ranidae: Amolops): a landscape genomics approach. *Zoological Journal of the Linnean Society*, 190, 65-78.

Cohen, M. P., & Alford, R. A. (1996). Factors affecting diurnal shelter use by the cane toad, Bufo marinus. *Herpetologica*, 172-181.

Cortés-Suárez, J. E. (2017). Uso de microhábitat por parte del sapo gigante *Rhinella horribilis* en pastizales en el municipio de Villa de Leyva, Boyacá, Colombia. *Revista Biodiversidad Neotropical*, 7(4), 253-257.

Giordano, A. R., Ridenhour, B. J., & Storfer, A. (2007). The influence of altitude and topography on genetic structure in the long-toed salamander (*Ambystoma macrodactulym*). *Molecular Ecology*, 16, 1625-1637.

Homola, J. J., Loftin, C. S., & Kinnison, M. T. (2019a). Landscape genetics reveals unique and shared effects of urbanization for two sympatric pool-breeding amphibians. *Ecology and Evolution*, 9(20), 11799-11823.

Huang, L., Li, J., Anboukaria, H., Luo, Z., Zhao, M., & Wu, H. (2016). Comparative transcriptome analyses of seven anurans reveal functions and adaptations of amphibian skin. *Scientific Reports*, 6(1), 24069.

Medina, R., Wogan, G.O.U. , Bi, K., Termignoni-García, F., Bernal, M.H., Jaramillo-Correa, J.P., Wang, I.J., & Vázquez-Domínguez, E. (2021). Phenotypic and genomic diversification with isolation by environment along elevational gradients in a neotropical treefrog. *Molecular Ecology*, 30, 4062-4076.

Murphy, M. A., Dezzani, R., Pilliod, D. S., & Storfer, A. (2010). Landscape genetics of high mountain frog metapopulations. *Molecular Ecology*, 19, 3634-3649.

Ramírez-Arce, D. G., Ochoa-Ochoa, L. M., & Lira-Noriega, A. (2022). Effect of landscape composition and configuration on biodiversity at multiple scales: a case study with amphibians from Sierra Madre del Sur, Oaxaca, Mexico. *Landscape Ecology*, 37(8), 1973-1986.

Rollins, L. A., Richardson, M. F., & Shine, R. (2015). A genetic perspective on rapid evolution in cane toads (*Rhinella marina*). *Molecular Ecology*, 24, 313-327.

Wells, K. D. (2019). The ecology and behavior of amphibians. University of Chicago Press, Chicago, USA.

Table S5. Hypotheses of multivariate models (environmental variables) that could potentially affect *Rhinella horribilis* connectivity. Models were tested separately for each landscape. P1O: landscape 1, P2O: landscape 2. Genetic distance (F_{ST} and *Dps*) was used as the independent factor. Environmental variable names in table S1.

**Model**	**Variables**	**Landscape**	**Parameters**	**Landscape**
Aquatic	TBW + TS	P1O	TBW + TS	P2O
Structural	NDVI + TS + TBW	P1O	NDVI + TS + TBW	P2O
Biological 1	NDMI + TBW + TS + RH	P1O	NDMI + TBW + TS + RH	P2O
Biological 2	AT + SR + EVA + BSI + ELV	P1O	AT + SR + BSI	P2O

Table S6. Paired genetic distance F_{ST} (below) and *Dps* (above) between sampling sites for *Rhinella horribilis* in landscape 1 (P1O) and landscape 2 (P2O). Sampling sites names in table S3.

P1O	AMA	AMB	CAA	CAB	IPA	IPB	ZAA	ZAB
**AMA**	0	0.0595	0.0606	0.0544	0.0608	0.0627	0.0688	0.0645
**AMB**	0.0203	0	0.0634	0.0624	0.0650	0.0620	0.0700	0.0665
**CAA**	0.0260	0.0276	0	0.0537	0.0647	0.0635	0.0664	0.0643
**CAB**	0.0200	0.0235	0.0088	0	0.0583	0.0594	0.0650	0.0598
**IPA**	0.0321	0.0296	0.0313	0.0269	0	0.0564	0.0679	0.0628
**IPB**	0.0220	0.0143	0.0193	0.0146	0.0078	0	0.0665	0.0638
**ZAA**	0.0386	0.0370	0.0300	0.0279	0.0339	0.0229	0	0.0529
**ZAB**	0.0364	0.0315	0.0286	0.0257	0.0318	0.0209	0.0007	0
**P2O**	**PNB**	**PNC**	**SAA**	**SAB**	**SCB**	**TEA**	**TEB**	
**PNB**	0	0.0793	0.0826	0.0714	0.0825	0.0862	0.0833	
**PNC**	0.0149	0	0.0854	0.0773	0.0772	0.0813	0.0738	
**SAA**	0.0386	0.0252	0	0.0605	0.0771	0.0901	0.0819	
**SAB**	0.0504	0.0312	0.0001	0	0.0775	0.0828	0.0793	
**SCB**	0.0198	0.0045	0.0125	0.0271	0	0.0789	0.0702	
**TEA**	0.0348	0.0145	0.0358	0.0434	0.0138	0	0.0671	
**TEB**	0.0213	0.0076	0.0274	0.0325	0.0098	0.0041	0	

Table S7. Optimized resistance values of each land use class based on individual generalized linear mixed models, using F_{ST} and *Dps* genetic distances for *Rhinella horribilis* in two landscapes (P1O and P2O) in Oaxaca, southern Mexico. Only P1O had importance based on the percentage of pseudo-bootstrap results, see Table 1.

**Class**	**Urban area (UA)**	**Crops and pastures (CP)**	**Secondary forest (SF)**	**Water bodies (WB)**	**Dense forest (DF)**	**Bare soil (BS)**
**Landscape 1 (P1O)**						
F_{ST}						
**Resistance values**	1	1.1397	374.7151	817.0233	2500	2053.5775
Dps						
**Resistance values**	1	1.1395	396.2236	1527.073	1222.0456	2500
**Landscape 2 (P2O)**						
F_{ST}						
**Resistance values**	1	2500	683.9606	14.1773	683.9606	177.5436
Dps						
**Resistance values**	1	664.8272	1327.6239	1122.1877	1327.6239	1.0224
