# Supplementary material for: Functional connectivity patterns of the Giant Toad Rhinella horribilis in anthropogenically modified landscapes
Source: PLoS One. 2025 Oct 15;20(10):e0319111. doi: 10.1371/journal.pone.0319111 (PMC12527146; doi:10.1371/journal.pone.0319111)
Supplement: S3 File — (PDF) [file pone.0319111.s003.pdf]

Functional connectivity patters of the giant toad *Rhinella horribilis* in anthropogenically modified landscapes

Gerardo J. Soria-Ortiz, Leticia M. Ochoa-Ochoa, Juan P. Jaramillo-Correa, Íñigo Martínez-Solano, Ella Vázquez-Domínguez

Supplementary methods

I. Filtering of the study-wide dataset (P1O and P2O together)

We performed two processes for selecting linkage disequilibrium (LD) and minor allele count (mac) filters. First, to decide the cutoff for LD, we started with a filtered study-wide dataset that included: allele balance (0.25-0.75); minDP=5; minQG=20; only biallelic loci; maximum missing data allowed 20% (--*max-missing*); maximum heterozygosity 0.5 (--*max-obs-het*); 2(x) mode depth=34; and minor allele count=3 (--mac). Based on this dataset (54,194 SNPs) we used plink1.9 and ran --r2 to obtain the allele count square root correlation (Hemstrong et al., 2024), using the parameters --ld-window 1000, --ld-window-kb 50, --ld-window-r2 0. Based on the LD decay plot obtained (Fig 1), we chose a string cut-off value of 10kb for the LD filter, for which we found most of the loci with an r2 value below 0.2.

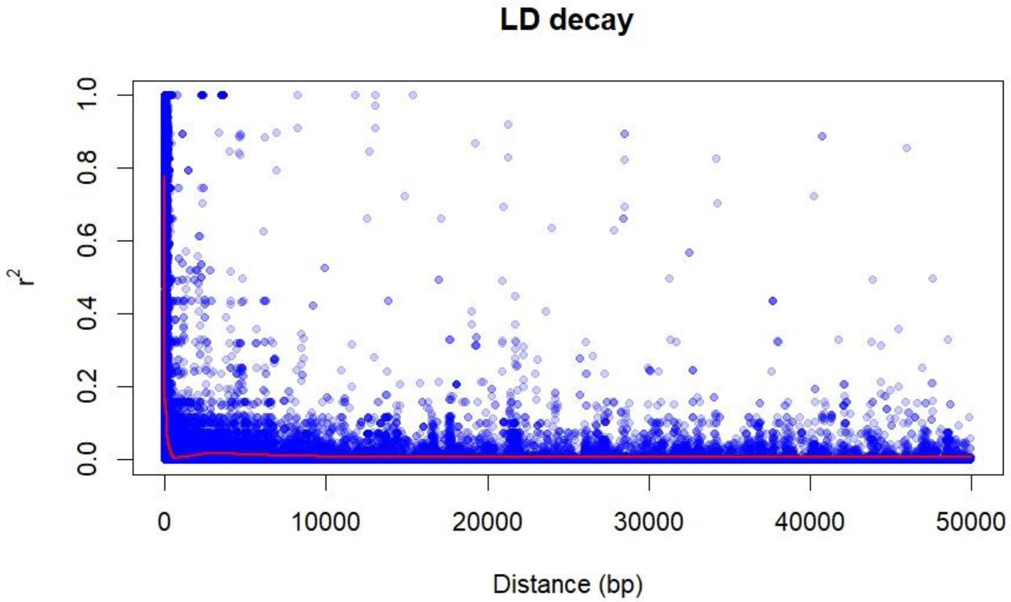

Fig 1. Linkage disequilibrium decay for the study-wide dataset (P1O and P2O; 190 samples). Blue dots indicate the r^2 of each SNP and the red line depicts the LD decay tendency. We observed that LD decayed at ≈ 2000 bp.

Second, to select the minor allele count (mac) filter for the study-wide dataset, we built a dataset with: allele balance (0.25-0.75); minDP=5; minQG=20; only biallelic loci; missing data 20%; linkage disequilibrium=10kb; maximum heterozygosity=0.5; 2(x) mode depth=34. Based on this dataset (29,414 before mac filter; Table 1), we created 8 datasets to select the best mac filter value: 3, 5, 7, 9, 11, 13, 15, 17, as recommended by Linck and Battey (2018). A --mac 3 filter indicates that the allele needs to be present at least in two individuals, while a --mac 17 at least in nine individuals. We ran a principal component analysis to identify which dataset recovered the highest structure (Fig 2; Table 1). Based on the PCA plots, we selected a --mac 7 filter that showed the clearest structure, while higher mac values did not improve it. This is consistent with Linck and Battey (2018) who indicate that in model-based population structure inference not only singletons, but all rare alleles, have a high noise to signal ratio, while common alleles accurately reflect expected relationships between individuals.

Table 1. *Rhinella horribilis* study-wide post-filtering process. The number of retained SNPs after each filter are shown in each column, in a step-by-step order. Column names are: allele balance (0.25 - 0.75 allele frequency); minDP: genotype depth; minGQ: genotype quality; only biallelic loci; max-missing: 20% of missing data allowed; thin: linkage disequilibrium 10kb; max-meanDP: site-mean-depth mode*2; max het: maximum observed heterozygosity allowed; mac: minor allele count. The mac column shows the different mac values tested in parenthesis. In bold, the final dataset used for the study-wide analyses.

**Initial**	**allele balance**	**minDP 5**	**minGQ 20**	**biallelic loci**	**max-missing 0.8**	**thin 10 kb**	**max-mean DP 34**	**max het 0.5**	**mac**
1134057	1134057	1134057	1134057	1134057	257654	29933	29414	27935	11929 (3)
									8243 (5)
									**6034 (7)**
									4563 (9)
									3515 (11)
									2786 (13)
									2216 (15)
									1784 (17)

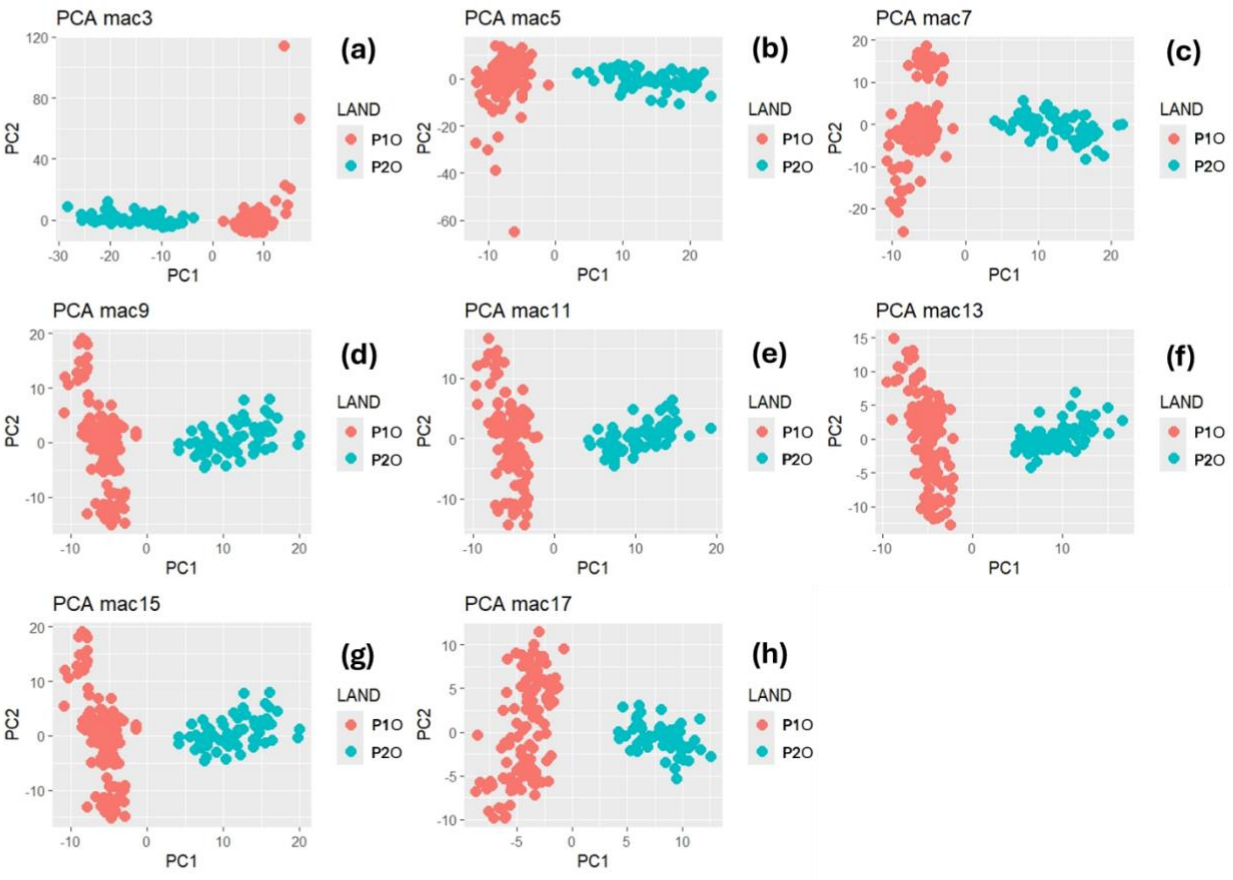

Figure 2. PCA plot of eight study-wide datasets from mac=3 to mac=17. mac=7 obtained the best structure between landscapes (P1O and P2O). Subsequent mac filters do not improve the results.

II. Filtering of the population-study datasets

For analyzing each landscape separately, we performed filtering by landscape and followed the same processes to select both LD and mac values. To this end, we first separated the unfiltered dataset into the two landscapes, P1O and P2O. Then, we built one dataset for each landscape (population-study) that included allele balance (0.25-0.75); minDP=5; minQG=20; only biallelic loci; missing data 20%; maximum heterozygosity=0.5; 2(x) mode depth=34, and mac=3, which yielded 37,682 SNPs for P1O and 22,840 for P2O. Next, we calculated r^2 and generated the LD decay plot. Based on these plots (Fig 3) we chose a string cut-off value of 10kb for the LD filter for both population-study datasets (P1O and OP2).

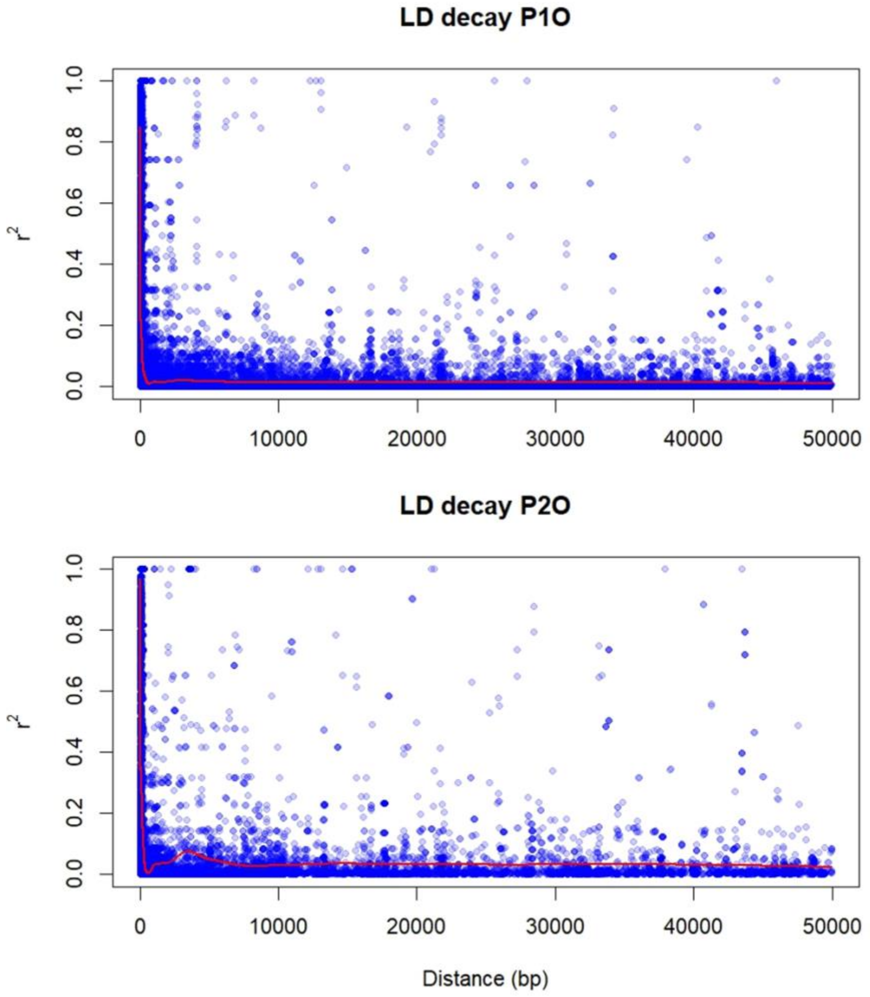

.

Fig 3. Linkage disequilibrium decay for the population-study datasets (P1O, 125 samples; P2O, 65 samples). Blue dots indicate the r^2 of each SNP and the red line depicts the LD decay tendency. We observed that LD decayed at ≈2000 bp for P1O and ≈5000 bp for P2O.

Next, to select the minor allele count (mac) filter for the population-study datasets, we built one more dataset for each landscape with the same filters described above, adding the 10kb LD filter in both landscapes. Likewise, using these two datasets (28,578 SNPs for P1O and 31,214 for P2O, pre mac filter; Table 2), we also created 8 datasets to select the best mac filter value: 3, 5, 7, 9, 11, 13, 15, 17 (Linck and Battey, 2018). We ran a PCA to identify which dataset recovered the best structure. Based on the PCA plots, we selected a --mac 7 filter that showed the clearest structure forP1O (Fig 4) and --mac 5 for P2O (Fig 5); higher mac values did not improve the results.

Table 2. *Rhinella horribilis* population-study post-filtering process by landscape (P1O and P2O). The number of retained SNPs after each filter are shown in each column, in a step-by-step order. Column names are: allele balance (0.25-0.75 allele frequency); minDP: genotype depth; minGQ: genotype quality; only biallelic loci; max-missing: 20% of missing data allowed; thin: linkage disequilibrium 10kb; max-meanDP: site-mean-depth mode*2; max het: maximum observed heterozygosity allowed; mac: minor allele count. The mac column shows the different mac values tested in parenthesis. In bold, the final dataset used for study-wide analyses.

P1O

**Initial**	**Allele balance**	**minDP 5**	**minGQ 20**	**biallelic loci**	**max-missing 0.8**	**thin 10 kb**	**max-mean DP 22**	**max het *0.5***	**mac**
**1134057**	1134057	1134057	1134057	1134057	278055	31539	29964	28578	9481 (3)
									5966 (5)
									**4088 (7)**
									2901 (9)
									2180 (11)
									1706 (13)
									1393 (15)
									1137 (17)

P2O

**Initial**	**Allele balance**	**minDP 5**	**minGQ 20**	**biallelic loci**	**max-missing 0.8**	**thin 10 kb**	**max-mean DP 24**	**max het 0.5**	**mac**
1134057	1134057	1134057	1134057	1134057	289244	33669	32469	31214	7362 (3)
									**4190 (5)**
									2679 (7)
									1832 (9)
									1352 (11)
									1050 (13)
									825 (15)
									653 (17)

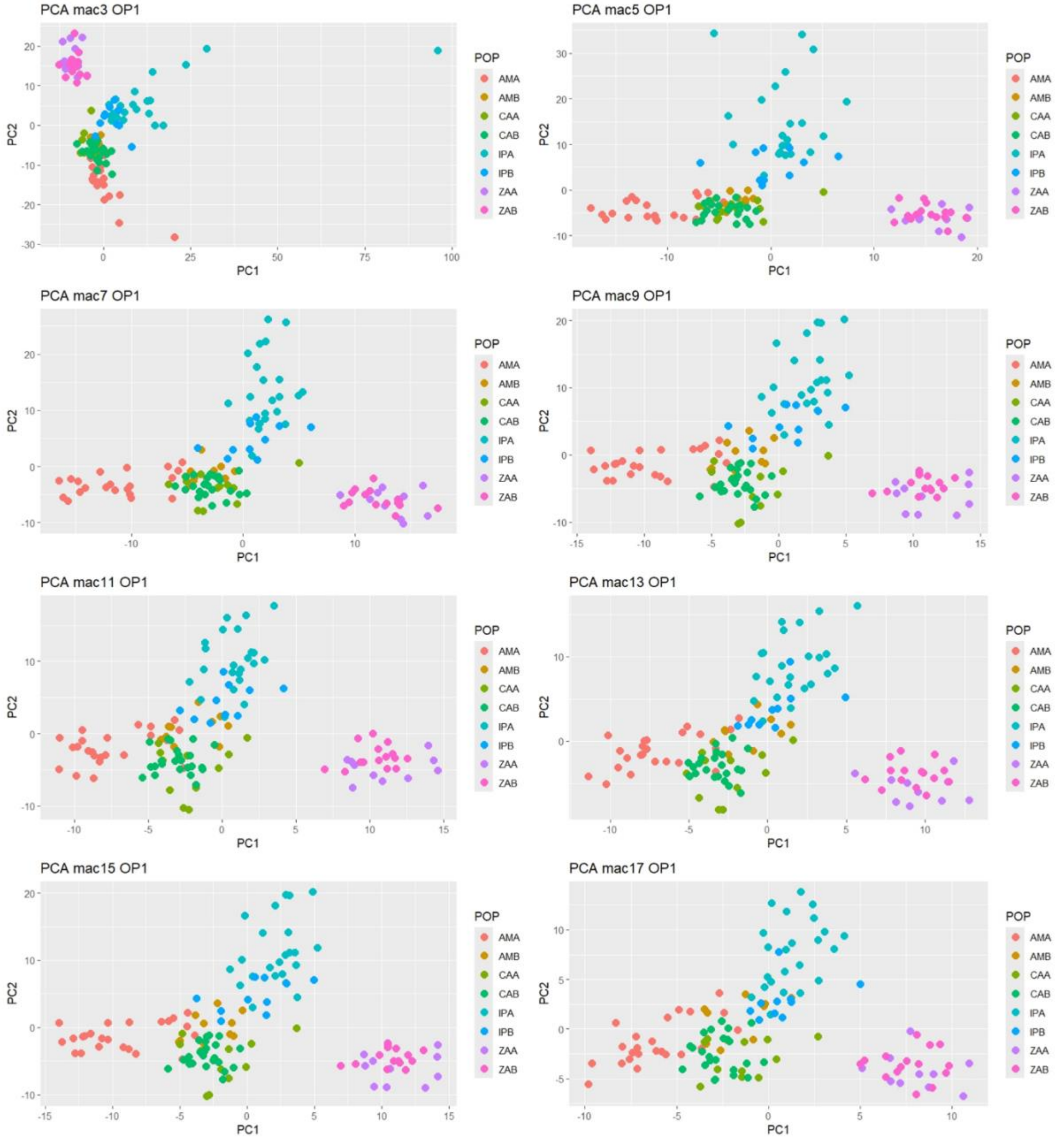

Figure 4. PCA plot of eight population-study datasets for landscape P1O from mac=3 to mac=17. mac=7 obtained the best structure; subsequent mac filters do not improve the structuring results.

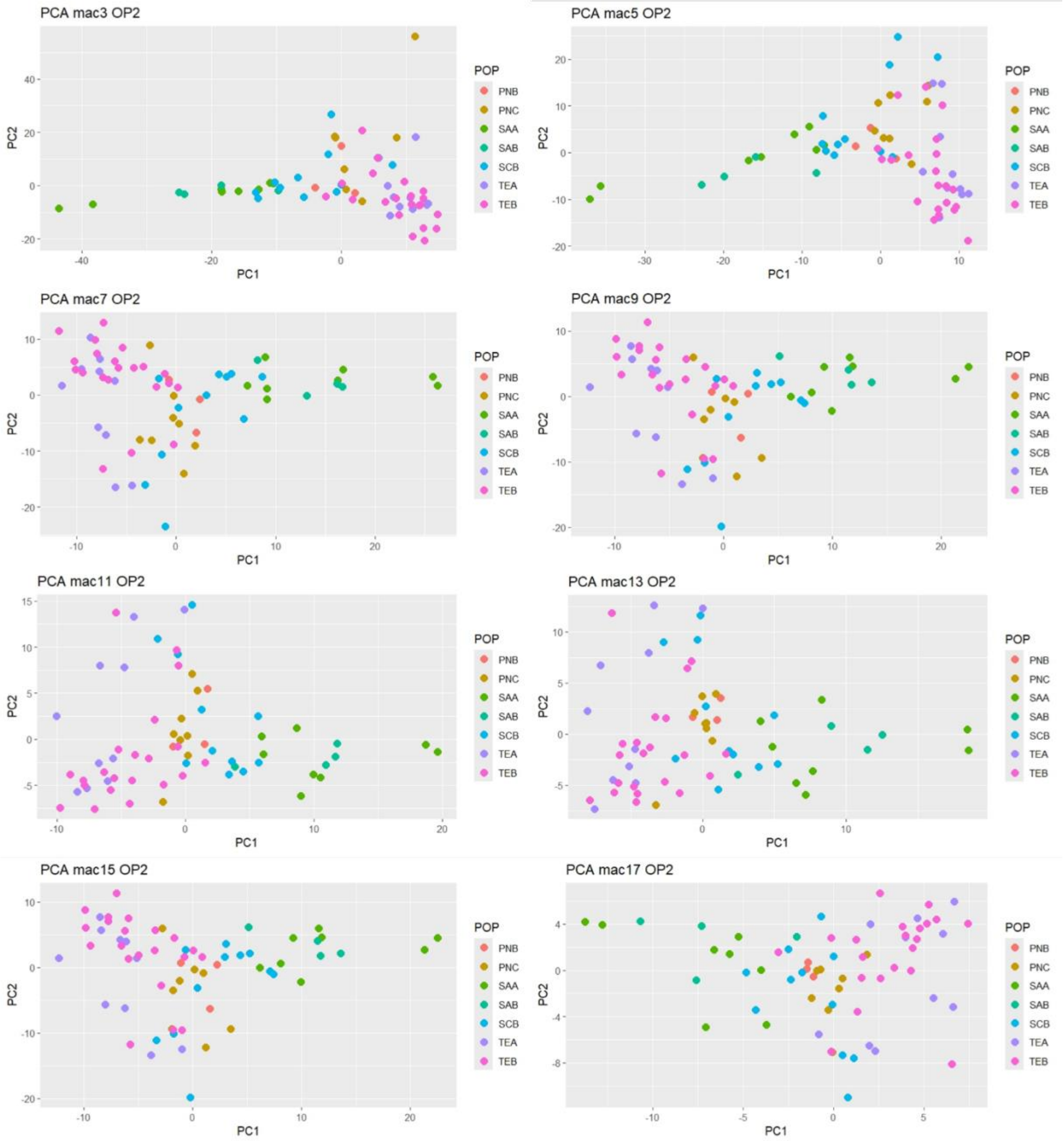

Fig 5. PCA plot of seven population-study datasets for landscape P2O from mac=3 to mac=17. mac=5 obtained the best structure; subsequent mac filters do not improve the structuring results.

R scripts used in the study compiled into five sections with the different analyses performed.

Contents:

SCRIPT 1 for genetic structure

SCRIPT 2 for genetic diversity comparisons between landscapes

SCRIPT 3 for barrier models

SCRIPT 4 for isolation by distance (IBD) tests

SCRIPT 5 for individual and multivariate resistance models

SCRIPT one

Script for genetic structure

Structure analyses are made for each landscape separately and for study-wide.

These scripts run when the work route has been previously established

This script is an example for P1O data

```
# Libraries

# Note: LEA and TESS packages have overlapping functions. To run an analysis, you must disable the other packages, and vice versa.

library(vcfR)

library(hierfstat)

library(adegenet)

library(PopGenReport)

library(ggplot2)

library(LEA)

library(tess3r)


# Import genomic, population and coordinates data.

VCF_P1_RH <- read.vcfR("P1O.vcf", verbose = FALSE) #vcf P1O, these can be changed for each vcf base data

pop_data_P1_RH <- read.table("popmap_P1O.txt") #popmap P1O, these can be changed for each popmap data

coor_P1_RH <- read.table("coord_id_P1O.txt") # Coord P1O at individual level. These can be changed for each coord data


# Calculation of genetic distance matrices

#Fst

genRHP1 <- vcfR2genind(VCF_P1_RH, return.alleles = TRUE) #Convert vcf to genind objet

genRHP1@pop <- as.factor(pop_data_P1_RH$V2) #Population information is added to genind object
```

P1_hier_RH <- genind2hierfstat(genRHP1) #Converts genind objects from adegenet into a hierfstat data frame

fst_P1_RH <- pairwise.neifst(P1_hier_RH, diploid = TRUE) #Estimate pairwise FSTs according to Nei (1987)

#Dps

Dps_P1_RH <- pairwise.propShared(genRHP1) #Calculates proportion of shared alleles

Dps_P1_RH_1 <- as.matrix(1-Dps_P1_RH) #Create Dps distance matrix

PCA analyses

P1RHgenl <- vcfR2genlight(VCF_P1_RH) # Convert vcfR objects to genlight

P1RHgenl@pop <- as.factor(pop_data_P1_RH$V2) # Population information is added to genind object

PCARHP1 <- glPca(P1RHgenl, nf = 50, scale = TRUE) # PCA run, nf = n umber of principal components to be retained

var_expl <- PCARHP1$eig / sum(PCARHP1$eig) # Extract proportion of explained variance

pc1_var <- round(var_expl[1] * 100, 2) #PCA 1

pc2_var <- round(var_expl[2] * 100, 2) #PCA 2

#Plot PCA

```
ggplot(data = data.frame(PCARHP1$scores), #PCA objet
 aes(x=PC1, y=PC2, col=P1RHgenl@pop)) + #popdata is specified
  geom_point(size = 4) +
  ggtitle("title") + #title name
  labs(
    x = paste0("PC1 (", pc1_var, "%)"), # proportion of explained variance is added
    y = paste0("PC2 (", pc2_var, "%)"),
    col = "POP"
  )+
  theme(
    plot.title = element_text(size = 20, face = "italic"),
    legend.title = element_text(size = 15),
```

```
  legend.text = element_text(size = 15),
  legend.key.size = unit(1, "cm"))

#DAPC analyses
#performs stratified cross-validation of DAPC
pramxp1RH <- xvalDapc(tab(P1RHgenl, NA.method = "mean"), # We use genlight objet
                pop(P1RHgenl), n.rep = 999)
#Run DAPC based on xvalDapc results
dapcP1RH <- dapc(P1RHgenl, n.pca=45, n.da = 7) # We use genlight object
#Plot DAPC
myCol <- c("tomato","firebrick4","chartreuse2","green4",
        "dodgerblue1","blue4","darkorchid1","purple4") #manual color palette
scatter.dapc(dapcP1RH, #DAPC objet
         leg=T,
         bg="white",
         scree.da=FALSE,
         pch=20,
         cell=2,
         cstar=1,
         col=myCol, # colors palet
         solid=.8,
         cex=4,
         clab=1,
         main ="title") #title name

#SNMF analyses
#The LEA library needs to be disabled.
project.snmf_P1_RH = snmf("P1O.geno", #Load the .geno file from the original route
                  K = 1:8, #number of populations
```

```
            ploidy = 2,
            entropy = TRUE,
            repetitions = 20,
            iterations = 100000,
            alpha = 100,
            seed=25, #This can be changed for independent runs
            project = "new")
#Plot to identify the best "K" with the lowest entropy value
plot(project.snmf_P1_RH, cex = 1.5, col = "red", pch = 19)
#Barplot
ce3_p1_RH <- cross.entropy(project.snmf_P1_RH, K=3)  #select the K value
best_3_p1_RH = which.min(ce3_p1_RH) #select best k repetition based on the lowest entropy value
my.colors3_p1_RH <- c("blue", "red", "green3") #manual palette colors
qmatrix3_p1_RH <- Q(project.snmf_P1_RH, #extract matrix of probability of assignation
          K = 3, run = best_3_p1_RH)
barplot(t(qmatrix3_p1_RH), col= my.colors3_p1_RH,
    border=NA, space=0, xlab="x-title",
    ylab="y-title",
    main = "title") #title name
axis(1, at = 1:nrow(qmatrix3_p1_RH), labels = pop_data_P1_RH$V2, las = 3, cex.axis = 1) #add
population labels


#TESS analyses
#The tess3r library needs to be disabled
tess3_p1_RH <- TESS3("P1O.geno", #Load the .geno file from the original route
          "coord_id_P1O.txt", #Load individual coordinates from the original route
          K=1:8, #number of populations
          ploidy = 2,
          entropy = TRUE,
```

```
                repetitions = 20,
                iterations = 1000000,
                alpha = 100,
                seed=20, #This can be changed for independent runs
                project = "new")
#Model evaluation and plotting is equal as SNMF plot


#SPCA analyses
#Build a connection network (CN)
Rh_graph_p1 <- chooseCN(genRHP1$other,  #individula coordinates are indicated
                 type=5,   #Neighbourhood by distance
                 d1=0,d2=0.22)  #saturated graph, depends of the distance between coordinates
RH.spca.p1 <- spca(genRHP1, xy=genRHP1@other, #run the SPCA analyses
             cn=Rh_graph_p1, nfposi=1,nfnega=1) #number of positive and negative eigenvalues
retained
#plot
colorplot(RH.spca.p1,cex=4, main="title")
```

```r
# SCRIPT two
# Script for genetic diversity differences between landscapes
# Statistical models are made one by one for each genetic metric
# Genetic diversity metrics were obtained by population function in stacks
# These scripts run when the work route has been previously established

# Libraries
library(nlme)

## Import base data
data <- read.csv("gen_div_SW_RH.csv", header = T)
data$Land <- as.factor(data$Land) #indicate landscape as a factor

#run the model
mod_spatial_He <- gls(He ~ Land, #change He for each genetic diversity.
            correlation = corGaus(form = ~ X_km + Y_km),
            data = data)
summary(mod_spatial_He) #evaluate the model
```

SCRIPT three

Script for barrier models

Barrier models were made one by one for each genetic matrix differentiation and landscape

These scripts run when the work route has been previously established

This script can be modified for the different landscape data.

This script is an example for P1O data

```
#Libraries
library(adegenet)
library(hierfstat)
library(vcfR)
library(PopGenReport)
library(ape)
library(vegan)
library(gdistance)
library(ecodist)

# Import base data
pop_data_P1 <- read.table("popmap_P1O.txt") # this can be changed for P2O popmap data
VCF_P1_RH <- read.vcfR("P1O.vcf", verbose = FALSE) #vcf P1O, this can be changed P2O vcf base data
bar_p1 <- read.csv("barriers_P1O.csv", header = TRUE) #barrier dummy variables for dbRDA, this can be changed P2O barrier base data
coord_p1 <-read.csv("coord_pop_P1O.csv", header = TRUE) #site/population level, this can be changed P2O coord base data

#Calculation of genetic distance matrices
gen_P1RH <- vcfR2genind(VCF_P1_RH, return.alleles = TRUE) #Convert vcf to genind objet
gen_P1RH@pop <- as.factor(pop_data_P1$V2) #Population information is added to genind object
#Fst
```

P1_hier <- genind2hierfstat(gen_P1RH) #Converts genind objects from adegenet into a hierfstat data frame

fst_P1 <- pairwise.neifst(P1_hier, diploid = TRUE) #Estimate pairwise FSTs according to Nei (1987)

fst_P1_M <- as.matrix(fst_P1) #create a Fst matrix

#Dps

Dps_P1 <- pairwise.propShared(gen_P1RH) #Calculates proportion of shared alleles

DPS_P1_M <- as.matrix(1-Dps_P1_RH)#create a Fst matrix

#PCOA for each genetic matrix

PCOAFstP1 <- pcoa(fst_P1_M)

PCOADpsP1 <- pcoa(DPS_P1_M)

vec_fst_P1 <- as.table(PCOAFstP1$vectors) #extrac vector of PCOA

vec_Dps_P1 <- as.table(PCOADpsP1$vectors)

#dbRDA analyses for each barrier

#Fst

RDA_Fst_barrier_P1 <- rda(vec_fst_P1 ~ bar_p1$Road1 + bar_p1$Road2 + bar_p1$Road3 + bar_p1$River1+ bar_p1$River2)

anova(RDA_Fst_R1_P1, permu=1000)

#DPS

RDA_DPS_barrier_P1 <- rda(vec_Dps_P1 ~ bar_p1$Road1 + bar_p1$Road2 + bar_p1$Road3 + bar_p1$River1 + bar_p1$River2)

anova(RDA_DPS_CA_P1, permu=1000)

#MRMM

#Prepare data

#This is an example of least-cost distance from a raster barrier .asc

coord_p1 <-SpatialPoints(coord_p1) #create objects of class SpatialPoints-class

bar_r1_p1 <- read.asciigrid("bar_r1_P1O.asc") #Read .asc objet. Here the raster needs be changed for each barrier

```
bar_r1_p1_r <- raster(bar_r1_p1) #Create a RasterLayer object

bar_r1_p1_r_t <- transition(bar_r1_p1_r, transitionFunction=mean, 8) #Create a Transition object

bar_r1_p1_r_t <- geoCorrection(bar_r1_p1_r_t, type = "c") #Correct 'TransitionLayer'

LC_BR1_p1 <- costDistance(bar_r1_p1_r_t, q2) #calculates the least cost distance

#Repit this processes for each barrier .asc layer


#Run the model

#FST

MRM_P1_FST <- MRM(as.dist(fst_P1) ~ as.dist(LC_BR1_p1) +

        as.dist(LC_BR2_p1) + as.dist(LC_BR3_p1) +

        as.dist(LC_BRiv1_p1) + as.dist(LC_BRiv2_p1), method = "linear", nperm = 10000)

summary(MRM_P1_FST) #evaluate the model

#DPS

MRM_P1_DPS <- MRM(1-Dps_P1_RH ~ as.dist(LC_BR1_p1) +

        as.dist(LC_BR2_p1) + as.dist(LC_BR3_p1) +

        as.dist(LC_BRiv1_p1) + as.dist(LC_BRiv2_p1), method = "linear", nperm = 10000)

summary(MRM_P1_DPS) #evaluate the model
```

SCRIPT four

```
# Script for isolation by distance (IBD)

# IBD were made one by one for each genetic distance and landscape

# These scripts run when the work route has been previously established

# This script can be modified for the different landscape data

# This script is an example for P1O data


#Libraries

library(vcfR)

library(hierfstat)

library(adegenet)

library(PopGenReport)

library(ade4)

library (fossil)


#Prepare data

geo_p1 <- read.table ("coord_pop_P1O.csv", header=T, sep=",") #Load coordinates at pop level

km_p1 <- earth.dist(geo_p1[,c("Lon","Lat")], dist = F) #Create a distance matrix

VCF_P1_RH <- read.vcfR("P1O.vcf", verbose = FALSE) #vcf P1O

pop_data_P1_RH <- read.table("popmap_P1O.txt") #popmap P1O



# Calculation of genetic distance matrices

#Fst

genRHP1 <- vcfR2genind(VCF_P1_RH, return.alleles = TRUE) #Convert vcf to genind objet

genRHP1@pop <- as.factor(pop_data_P1_RH$V2) # Population information is added to genind
object

P1_hier_RH <- genind2hierfstat(genRHP1) #Converts genind objects from adegenet into a hierfstat
data frame
```

```
fst_P1_RH <- pairwise.neifst(P1_hier_RH, diploid = TRUE) #Estimate pairwise FSTs according to Nei (1987)

#Dps

Dps_P1_RH <- pairwise.propShared(genRHP1) #Calculates proportion of shared alleles

Dps_P1_RH <- as.matrix(1-Dps_P1_RH) #Distance matrix Dps


#two dimension IBD

linearized_Fst_RHp1 <- fst_P1_RH / (1 - fst_P1_RH) #linarized Fst

linearized_Dps_RHp1 <- Dps_P1_RH / (1 - Dps_P1_RH) #linarized Dps

log_geo_km1pRH <- log(km_p1)

fst_p1RH <- linearized_Fst_RHp1[upper.tri(linearized_Fst_RHp1)] #extract upper Fst values

Dps_p1RH <- linearized_Dps_RHp1[upper.tri(linearized_Dps_RHp1)] #extrac upper Dps values

km_p1RH <- log_geo_km1pRH[upper.tri(log_geo_km1pRH)] #extrac km dist values

#model

ibd_rhp1 <- lm(fst_p1RH ~ km_p1RH)

summary(ibd_rhp1) #evaluate

ibd_rhp1_Dps <- lm(Dps_p1RH ~ km_p1RH)

summary(ibd_rhp1_Dps) #evaluate the model

#plot

plot(as.vector(log_geo_km1pRH), as.vector(linearized_Fst_RHp1),# change linearized_Fst_RHp1 for linearized_Dps_RHp1

   xlab = "x-name", ylab = "y-name",

   main = "title",

   pch = 16, col = "blue",

   ylim = c(0, 0.05))

lineal <- lm(fst_p1RH ~ km_p1RH )

abline (lineal, col="red", lwd=5)#add tendency line
```

SCRIPT five

Script for individual and multivariate resistance models

Resistance models were made one by one for each environmental layer, genetic distance and landscape

These scripts run when the work route has been previously established

This script can be modified for the different landscape data.

This script is an example for P1O data

```
#Libraries

library(ResistanceGA)

library(vcfR)

library(hierfstat)

library(adegenet)

library(PopGenReport)


#prepare data

VCF_P1_RH <- read.vcfR("P1O.vcf") #vcf P1O,

gen_p1 <- vcfR2genind(VCF_P1_RH, return.alleles = TRUE) #Convert vcf to genind objet

popmap_p1 <- read.table("popmap_P1O.txt")  #popmap P1O

gen_p1@pop <- as.factor(popmap_p1$V2) # Population information is added to genind object

samples1 <- read.table("coord_pop_P1O.csv", header = TRUE) #load coordinates pop level

samples_op1 <- SpatialPoints(samples1[,c(2,3)]) #create objects of class SpatialPoints-class


#genetic distance

p1_hier <- genind2hierfstat(gen_p1) #Converts genind objects from adegenet into a hierfstat data frame

fst_P1 <- pairwise.neifst(p1_hier, diploid = TRUE) #Estimate pairwise FSTs according to Nei (1987)

Dps_P1_RH <- pairwise.propShared(gen_p1dps) #Calculates proportion of shared alleles

Dps_P1_RH <- as.matrix(1-Dps_P1_RH) #Distance matrix Dps
```

```
#Run resistance model univariate

#You need to run this function layer by layer, in total 10 environmental layers

#Import raster data

BSI <- raster("bsi_P1O.asc") #load .asc layers. Here its necessary load layer by layer

outputp1BSI <- "output_folder" #absolute path

# Optimización Resistance GA

GA.input.BSI.p1 <- GA.prep(ASCII.dir= BSI, #raster to evaluate

            Results.dir= outputp1BSI, #output folder

            method = "AIC", #method to evaluate the model

            select.trans = list("A"), # "A" refer to all equation transformation (monomolecular and
Ricker). "NA" if it's a categorical variable

            max.cat = 2500, #if is a categorical variable, then you need to use the argument
min.cat=1

            maxiter = 100, #max number of iterations for the model

            run = 25) #Number of consecutive generations without any improvement before the GA
is stopped

# Load genetic data

gdist.inputp1 <- gdist.prep(n.Pops = length(samples_op1), # number or populations

              samples = samples_op1, # Provide a SpatialPoints-class

              response = lower(fst_P1), #Genetic distance. Here needs change for "response =
lower(Dps_P1_RH_2)"

              method = 'costDistance') #Least cost distance calculation

# Run optimization

SS_results_BSI_P1_FST <- SS_optim(gdist.inputs = gdist.inputp1,

                GA.inputs = GA.input.BSI.p1)

#save the SS_results as a .rda, you will need this to run boostrap

save(SS_results_BSI_P1_FST, file = "output_folder/name.rda")#absolute path


#Run resistance multivariate models

#Import spatial map data layer by layer
```

```
kriosp1 <- raster("ks_P1O.asc") #example streams. Here needs to load individual layer one by one

#Build RasterStack by multivariate model
#acuatic model p1, TBW + TS
AcuaticModel_p1 <- stack(kriosp1, kpozasp1)
#Structural model p1, NDVI + TS + TWB
Structuralmodel_p1 <- stack(kriosp1, kpozasp1, ndvip1)
#Biological model 1 p1, NDMI + TBW + HR + TS
Biomodel1_p1 <- stack(ndmip1, kpozasp1, kriosp1, hrp1)
#Biological model 2 p1, BSI + AT + SR
Biomodel2_p1 <- stack(bsip1, tempp1, radp1)

#you need run this function for each multivariate model
multilayerACUMOD_p1 <- AcuaticModel_p1
outputACUMODp1 <- "output_folder" #absolute path
# Optimización Resistance GA
GA.inputs.multiACUMODp1 <- GA.prep(ASCII.dir= multilayerACUMOD_p1,
                    Results.dir= outputACUMODp1,
                    method = "AIC",
                    maxiter = 100,
                    run = 25)
# load genetic data, same as univariate models
# gdist.prep It is the same one used for multivariate models
# Run optimization multivariate model whit MS_optim
SS_results_ACUMOD_p1_FST <- MS_optim(gdist.inputs = gdist.inputp1,
                        GA.inputs = GA.inputs.multiACUMODp1)
#save the MS_results as a .rda, you will need this to run boostrap
save(SS_results_ACUMOD_p1_FST, file = "output_folder/ss_results_name.rda")#absolute path
```

```
#for bootstrap process you need to have previously run all the environmental and categorical layers
#and save the .rda objects.

#bootstrap analysis from previous optimized surfaces

fst_p1_rh <- as.matrix(dist(fst_P1)) #as matrix

colnames(fst_p1_rh)<- NULL

rownames(fst_p1_rh)<- NULL

#note: same process for Dps matrix

#load one by one important ss_results of individual and multivariate models

load("path/ss_results_name.rda") #absolute path

modBSIfstp1 <- SS_results_BSI_P1_FST

# Extract relevant components from SS_results or MS_results object one by one in a list

cd.list <- c(modBSIfstp1$cd,  modNDVIfstp1$cd, ...)#cost distance of each layer

k.list <- rbind(modBSIfstp1$k, modNDVIfstp1$cd, ...)#number of parameters of each layer

#run bootstrap

Boostrap_p1_Fst <- Resist.boot(mod.names = names(cd.list),

                    dist.mat = cd.list,

                    n.parameters = k.list[,2],

                    sample.prop = 0.8, #Proportion of observations to be sampled each iteration

                    iters = 10000,

                    obs = 8, #Total number of observations (populations)

                    genetic.mat = fst_p1_rh) #genetic matrix

#Saving bootstrap results

write.csv(Boostrap_p1_Fst,  file = "output_folder/Boostrap_name.csv")#absolute path
```
