# Supplementary material for: Functional connectivity patterns of the Giant Toad Rhinella horribilis in anthropogenically modified landscapes
Source: PLoS One. 2025 Oct 15;20(10):e0319111. doi: 10.1371/journal.pone.0319111 (PMC12527146; doi:10.1371/journal.pone.0319111)
Supplement: Attachment — Submitted filename: comments_to_authors.pdf [file pone.0319111.s004.pdf]

Soria-Ortiz et al. (PONE-D-25-03029) present a landscape genomics study of the giant toad (*Rhinella horribilis*) in two study areas in Mexico. A primary goal of the study is to identify signatures of spatially varying selection associated with environmental variation and test whether these signatures are concordant or discordant between the two study areas. The authors find some concordant signatures of selection, evident in the same candidate SNPs identified as associated with the same environmental factors in both study areas. Although this manuscript is within the scope of *PLoS One*, I have two major concerns that motivated me to recommend rejection of the manuscript in its current form. If revised appropriately, the manuscript would be suitable for publication in *PLoS One*. These two major concerns are the following:

1) The authors appear to have omitted key genotype and SNP filters routinely applied in population genomics. The authors also implemented a Hardy Weinberg Equilibrium filter that is well-known for distorting inference of population genetic differentiation, which is the focus of approximately half of the manuscript. My line comments explain these issues in greater detail. Correcting potential deficiencies in data filtering could substantially alter the results.

2) I believe that the central question asked in the manuscript – that is, whether genomic signatures of local adaptation are repeated in two different study areas – is not addressed with the sophistication offered by recently developed methods. For example, the genome-wide repeatability analysis of Yeaman et al. (2018) or the genome scan for repeatability at individual loci introduced in Booker et al. (2023; full references provided in the relevant line comment below) were specifically designed to address this question; these methods have been validated using simulated data. The authors did not use either of these methods. Instead, the authors used an *ad hoc* method with unknown reliability.

My line comments (below) elaborate on the aforementioned major concerns and indicate more minor feedback throughout the manuscript. Relevant lines (L) are indicated at the start of each comment.

Data availability: I note that although the authors intend to release a VCF file containing individual genotypes, the authors do not indicate a mechanism for publicly releasing their raw sequencing data (i.e., demultiplexed raw sequencing reads). The raw data should be released so that alternative genotyping methods could be used if other researchers wish to use these data. Additionally, the authors do not indicate a mechanism for publicly releasing the scripts used to analyze their data; written descriptions of methods in the main text and/or supporting information are often not sufficient for exactly replicating analyses. I recommend that the authors include their analysis scripts in their Dryad repository and/or in a GitHub repository.

L68-69: The homogenizing effect of gene flow does not imply that alleles experiencing spatially varying selection will be *lost*. It suggests that locally adaptive genetic differentiation may not evolve because selection may be ineffective compared to gene flow.

L75-77: these landscape factors likely do not universally decrease functional connectivity across all species. Revising this to state "In amphibians, some landscape factors *often* negatively affect…" would temper this claim. However, I am not sure this extended discussion of species-specific environmental effects is warranted in the Introduction.

L123: I find the description of the two study areas as distinct landscapes a bit misleading. The two study areas are separated by 70km but occur on the same landmass. More importantly, the authors have presupposed that the samples collected in the two study areas are genetically distinct units that should be analyzed separately. It is possible that the samples in the two study areas represent distinct genetic clusters; however, this hypothesis is not explicitly tested in the authors' population structure analyses, as they carried out genetic clustering algorithms within each study area separately. Grouping sampling localities for further analysis should be motivated by biology (e.g., genetic clusters), not based on arbitrarily defined geographic units. My suggestion here is to 1) first run genetic clustering algorithms at the total dataset level (i.e., both study areas analyzed collectively); and 2) group localities by high-level genetic clusters for further analyses.

L144: Inserting the figure legends throughout the manuscript (and separated from the actual figures) made reviewing the figures challenging. In the future, I recommend that the authors either 1) insert both the figures *and* their corresponding legends throughout the manuscript; or 2) have all figure legends and figures at the end of the manuscript.

L151-173: The relationship between the environmental variables and human modification of habitat are not clearly explained. This is surprising given that the title of the manuscript indicates that the genomic signatures of local adaptation are associated with "anthropogenically modified landscapes." The environmental factors that were included (e.g., NDVI, water properties) are expected to vary spatially in the absence of anthropogenic activity, such that the connection between landscape genomic patterns and anthropogenic activity is unclear. The importance of human landscape modification could be more explicitly investigated by including some index of anthropogenic activity (e.g., local percent urban landcover; percent agricultural landcover) in the landscape resistance analyses and genotype-environment association (GEA) analyses. Perhaps the authors can more clearly explain how their chosen environmental factors correlate with anthropogenic activity.

L151-173: The use of *in situ*, contemporary measurements of environmental variation is welcome, but it is not clear whether the timeframe of measurement is most relevant to selection. The authors state that sampling was conducted in the rainy season of 2019, so I assume here that environmental measurements were taken in the same time period; the authors should clarify whether this is true. This means that selective pressures that might occur at other time periods may be missed. For example, amphibians are generally prone to desiccation, such that variables like maximum annual temperature could be relevant. Additionally, natural selection is a cumulative process, such that longer-term environmental trends might be relevant but not

captured by measurements taken over a short time interval. Additional environmental factors can be sourced from public databases such as WorldClim 2.

L176: The manuscript states that in total, 190 samples were subject to DNA extraction. How many samples were from landscape 1, and how many were from landscape 2? Given that analyses were conducted independently for the two study areas, the per-landscape sample size is the more important than the total sample size.

L180: Why was only 5% of the reference genome used for *in silico* restriction enzyme digestion? Were the results of this fraction of the genome extrapolated to the whole genome to predict the number of RAD loci that would be obtained?

L184: please report the total number of reads generated from sequencing as well as the per-sample sequencing depth.

L193-203. I note that the authors do not appear to have used genotype depth, genotype quality, or allele balance filters. Genotype depth filtering is distinct from what appears to be a per-SNP mean depth filter (indicated in L198); genotype depth filtering recodes individual genotypes to missing data when the genotypes are supported by too few reads. Typical thresholds include recoding genotypes to missing when their depth is <5x or <10x. A genotype depth filter is particularly important here because ddRAD protocols generally preclude the identification and removal of PCR duplicates, such that effective depth and thus genotype quality may be overestimated (see Andrews et al. 2016 for more discussion). Genotype quality filters will similarly remove low-confidence genotypes; a threshold of GQ < 20 or GQ < 30 is typical. Allele balance (AB) refers to evenness among reads supporting the two different alleles present in heterozygous genotypes. Because the expected value of AB is 0.50, typical AB thresholds include recoding heterozygous genotypes to missing when AB is outside the following range: $0.25 \leq AB \leq 0.75$. These three filters can be implemented in the R package SNPfiltR (DeRaad et al. 2022; full citation below). See also O' Leary et al. (2018) for greater discussion of these filters. These filters will increase the amount of missing data in the genotype matrix and should therefore be used *before* removing individuals and/or SNPs based on missing data. Without any information about sequencing depth, as I commented above, it is unclear whether the dataset may contain many low-quality genotypes that could affect inference of population structure and signatures of selection.

Andrews, K. R., Good, J. M., Miller, M. R., Luikart, G., & Hohenlohe, P. A. (2016). Harnessing the power of RADseq for ecological and evolutionary genomics. *Nature Reviews Genetics*, *17*(2), 81–92. https://doi.org/10.1038/nrg.2015.28

DeRaad, D. A. (2022). snpfiltr: An R package for interactive and reproducible SNP filtering. *Molecular Ecology Resources*, *22*(6), 2443–2453. https://doi.org/10.1111/1755-0998.13618

O'Leary, S. J., Puritz, J. B., Willis, S. C., Hollenbeck, C. M., & Portnoy, D. S. (2018). These aren't the loci you're looking for: Principles of effective SNP filtering for molecular ecologists. *Molecular Ecology*, *27*(16), 3193–3206. https://doi.org/10.1111/mec.14792

L193-203: I also note that SNPs do not appear to have been filtered for linkage disequilibrium (LD). This is most pertinent to the more stringently filtered dataset intended for detecting population structure. Many population genetic clustering algorithms assume that loci are independent, which may be violated here. I recommend determining the physical extent of substantially elevated LD and thinning SNPs based on that interval. E.g., if LD is substantially higher than background until a distance of 100Kb, the dataset should be thinned to retain one SNP per 100Kb; this can be implemented in VCFtools using the *thin* command. The larger dataset used for detecting signatures of selection does not need to be thinned for LD because statistics from multiple nearby SNPs can be used to boost the signal of selection (e.g., using the weighted Z analysis or WZA; Booker et al. 2024).

Booker, T. R., Yeaman, S., Whiting, J. R., & Whitlock, M. C. (2024). The WZA : A window-based method for characterizing genotype–environment associations. *Molecular Ecology Resources*, *24*(2), e13768. https://doi.org/10.1111/1755-0998.13768

L193-203: I also note that the authors do not appear to have implemented filters that seek to remove SNPs that are at high risk of representing collapsed paralogous sequences. There are two commonly used filters to do this: 1) a per-SNP observed heterozygosity filter (not the same as the HWE filter used by the authors); and 2) a per-SNP mean sequencing depth filter. Point #1 involves removing SNPs with unusually high observed heterozygosity in the total dataset (i.e., among all individuals from all localities). Typically, SNPs with observed heterozygosity > 0.50 are removed. This is because the maximum expected heterozygosity for a biallelic SNP in a panmictic population is 0.50 (occurring when the two alleles are equally frequent); the existence of population structure would make observed values > 0.50 even more unusual. SNPs that have a high observed heterozygosity in the total dataset are likely to represent collapsed paralogous sequences, which should be removed. For Point #2, SNPs with unusually high mean sequencing depth across individuals may also represent collapsed paralogous sequences. Per-SNP mean sequencing depth can be output using the command --site-mean-depth in VCFtools; one rule of thumb is to remove SNPs that have a mean sequencing depth greater than two times the modal value (Willis et al. 2017).

O'Leary, S. J., Puritz, J. B., Willis, S. C., Hollenbeck, C. M., & Portnoy, D. S. (2018). These aren't the loci you're looking for: Principles of effective SNP filtering for molecular ecologists. *Molecular Ecology*, *27*(16), 3193–3206. https://doi.org/10.1111/mec.14792.

Willis, S. C., Hollenbeck, C. M., Puritz, J. B., Gold, J. R., & Portnoy, D. S. (2017). Haplotyping RAD loci: An efficient method to filter paralogs and account for physical linkage. *Molecular Ecology Resources*, *17*(5), 955–965. https://doi.org/10.1111/1755-0998.12647

L194: "each one" meaning each landscape? Please clarify.

L198: It is not clear what “minimum depth of 10 samples to recognize a SNP as true” means. Does this mean that the mean depth across samples for a given SNP had to be $\geq$ 10 reads? Additionally, please replace “recognize a SNP as true” to “retain a SNP,” which makes no claim about whether the SNP is likely a real polymorphism.

L199-203: MAF is the abbreviation for minor allele frequency, not minimum allele frequency. The MAF threshold of 0.05 for tests for selection is consistent with best practices highlighted in Ahrens et al. (2021) and is fine. However, the MAF threshold of 0.10 for population structure analyses runs counter to best practices identified in the literature, where SNPs with rare but not singleton alleles should be kept to improve assignment accuracy (e.g., Linck and Battey 2019). A minor allele count threshold of 3 retains rare but not singleton alleles; this can be implemented in VCFtools using the command - *-mac 3*.

Linck, E., & Battey, C. J. (2019). Minor allele frequency thresholds strongly affect population structure inference with genomic data sets. *Molecular Ecology Resources*, *19*(3), 639–647. https://doi.org/10.1111/1755-0998.12995

L200-201: The use of a HWE filter is problematic because population structure *creates* deviations from HWE (the Wahlund effect). Removing loci showing strong deviations from HWE can distort estimates of population genetic differentiation. See Pearman et al. (2022; full citation below).

Pearman, W. S., Urban, L., & Alexander, A. (2022). Commonly used Hardy–Weinberg equilibrium filtering schemes impact population structure inferences using RADseq data. *Molecular Ecology Resources*, *22*(7), 2599–2613. https://doi.org/10.1111/1755-0998.13646

L205-226: I note that all of the genetic clustering methods used by the authors assume discrete genetic groups. Even TESS, which incorporates spatial information, assumes that allele frequencies are spatially homogenous within each genetic cluster. It is clear from Figure S6 that both study areas show a strong pattern of isolation by distance (IBD), suggesting that this species is better characterized as a spatially continuous population rather than a set of discrete genetic groups. A consequence of IBD is that discrete clustering algorithms will tend to overestimate the number of genetic groups in the data (e.g., see Fig 2 in Bradburd et al. 2018; full citation below). I recommend implementing the analysis ConStruct (Bradburd et al. 2018), which implements both a non-spatial, discrete genetic clustering model (similar to ADMIXTURE) and a spatially explicit genetic clustering model. Essentially, the spatial model explains observed genetic variation as reflecting the contributions of multiple “geographic-genetic layers” that are interpreted similarly to genetic clusters in discrete algorithms. However, in contrast to non-spatial discrete models, the spatial model accommodates greater biological realism by allowing genetic similarity to decay with geographic distance within each geographic-genetic layer. ConStruct also provides a way to explicitly compare the predictive performance of its non-spatial

and spatial models (i.e., model selection). Given that the authors are studying spatial patterns, a spatially explicit analysis such as ConStruct would perhaps yield inferences of population structure that better reflect reality.

Bradburd, G. S., Coop, G. M., & Ralph, P. L. (2018). Inferring Continuous and Discrete Population Genetic Structure Across Space. *Genetics*, *210*, 33–52.

L207: I would like to hear from the authors whether there might be some value to identifying population genetic clusters and describing population genetic differentiation at the total dataset level (i.e., by running analyses using all individuals from the two study areas collectively). Currently, the manuscript presupposes that the two study areas are genetically distinct units that should be analyzed separately. However, they occur in the same landmass just 70km apart, and weak differentiation within each study area (max FST ~ 0.03) suggests that geographic boundaries between genetic clusters may not align with the study areas (e.g., some localities in landscape 2 might actually assign to genetic clusters in landscape 1 or vice versa), in which case the dataset should be subdivided by genetic cluster rather than arbitrarily defined landscape areas. Including population structure analyses at the total dataset level might also give some context to the identification of repeated signatures of genetic-environment associations. E.g., if there is only weak genetic differentiation between the two study areas, it may be that the genetic clusters in the two study areas have adapted to repeated environmental gradients from shared standing variation.

L214: The authors have applied DAPC using groups of samples defined *a priori* (i.e., the sampling localities). It is also possible to use K-means clustering to objectively define genetic clusters based on the genetic data and follow that with DAPC; i.e., using the function find.clusters and following it with the typical DAPC workflow. I bring attention to this because it is not clear what the purpose of DAPC here is; if it is to cluster samples genetically, then the objective K-means workflow should be used.

L328: Which correlation coefficient was used (E.g., Pearson's, Spearman's, Kendall's)?

L329: I note that lfmm_ridge is one implementation of *LFMM 2*, not the original LFMM that is cited here as reference 74. The appropriate reference for LFMM 2 is Caye et al. (2019; full citation below).

Caye, K., Jumentier, B., Lepeule, J., & François, O. (2019). LFMM 2: Fast and Accurate Inference of Gene-Environment Associations in Genome-Wide Studies. *Molecular Biology and Evolution*, *36*(4), 852–860. https://doi.org/10.1093/molbev/msz008

L324: RDA models can be used to evaluate variance inflation factors (VIFs) and remove highly correlated environmental factors. This process is different from evaluating pairwise correlations between environmental factors (which the authors have done, as noted in L319) because VIFs assess how multicollinearity among predictors leads to instability in model parameter estimation.

I recommend that the authors use a VIF-based environmental factor thinning procedure for their RDA (e.g., remove environmental factors with VIF > 10).

L326: How many permutations were used for anova.cca?

L333: The authors state that they "… ran *these* analyses with K=3." Which analyses are being referred to? I note that earlier in this paragraph, the authors describe RDA, which does not account for population structure; if the authors accounted for population structure in RDA, they should describe the use of a partial RDA (pRDA) procedure.

L334: The use of a multiple test correction and choice of significance threshold are not indicated for LFMM.

L338: How were candidate SNPs assigned to genes? Proximity?

L357-376: It is not clear what the authors mean by testing one study area's candidate SNPs "in the other landscape." Figure S3 does not clarify this.

L376: There is a lack of detail regarding the use of Fisher's exact test. E.g., were multiple tests done for different environmental factors?

L357-375: I note that there are specialized methods for detecting repeated signatures of selection among multiple lineages, none of which are used in this manuscript. E.g., the C-score method of Yeaman et al. (2018) takes as input the test statistic associated with *every SNP* for two or more lineages and determines whether the genome-wide test statistic values are more similar between the lineages than expected by chance. This could be done multiple times for each environmental factor. To run this analysis, the authors will have to subset the SNPs to include only those present in both study areas. Alternatively, the authors could first aggregate per-SNP statistics into genomic windows using a method like the weighted Z analysis (WZA; Booker et al. 2024) so that SNPs only identified in one study area can still contribute to inference; the C-score analysis can then be run on genomic window-level statistics. I note also that Booker et al. (2023) recently introduced a formal genome scan for repeated evolution; this method can identify signatures of repeated evolution at individual loci rather than the genome-wide inference provided by the C-score analysis. Both methods are relevant here. Full citations for these references are given below.

Yeaman, S., Gerstein, A. C., Hodgins, K. A., & Whitlock, M. C. (2018). Quantifying how constraints limit the diversity of viable routes to adaptation. *PLOS Genetics*, *14*(10), e1007717. https://doi.org/10.1371/journal.pgen.1007717

Booker, T. R., Yeaman, S., Whiting, J. R., & Whitlock, M. C. (2024). The WZA : A window-based method for characterizing genotype–environment associations. *Molecular Ecology Resources*, *24*(2), e13768. https://doi.org/10.1111/1755-0998.13768

Booker, T. R., Yeaman, S., & Whitlock, M. C. (2023). Using genome scans to identify genes used repeatedly for adaptation. *Evolution*, *77*(3), 801–811. https://doi.org/10.1093/evolut/qpac063

L379: I note that the SNP dataset has 190 total samples, which was the number of individuals that were initially sequenced. L200 states that individuals with >20% missing data were removed. In practice, were any individuals removed? I.e., are the 125 landscape 1 and 65 landscape 2 individuals the final, post-filtering sample sizes for the two landscapes?

L379: I commented on this earlier, but the per-landscape sample sizes should be indicated in the Methods near L176 where the total sample size is first reported. The per-landscape sample sizes are most important given that analyses were conducted independently in the two landscapes/study areas.

L380: The reduction from ~4.6M "raw" SNPs to ~10K is not unusual for RAD data, but it would be informative to have a supplementary table that indicates the number of SNPs retained after each filtering step.

L380-382: Calling the more stringently filtered dataset "neutral data" is potentially misleading. It would be clearer to call this the "more stringently filtered dataset used for population structure analyses." Similarly, the less stringently filtered dataset ("full data") would more clearly be described as the dataset used for GEA analyses. I also note that the authors do not describe removing candidate SNPs from the "neutral" dataset, which further calls into question this terminology.

L381-384: "Average coverage" is not meaningful. Is this referring to the mean across SNPs, across individuals, or both?

L384: What were the final statistics regarding missing data? I.e., mean and range of missing data per SNP, mean and range of missing data per individual?

L387: The Methods section does not describe the statistical test for comparing genetic diversity between the two study areas. It looks like a t-test may have been used, which should be described in the Methods. Importantly, the authors should confirm whether the assumptions of the t-test were met; this process is also not described. Additionally, what was the unit of observation – locality?

L401-403: This sentence strikes me as interpretation, which should not occur in the Results section.

L423: why do the R-squared estimates and p-values indicated here differ from those shown in Figure S6?

L430-454: Please include the "%" symbol after each R-squared value.

L431: Please indicate that "85.6%" refers to the percent of pseudo-bootstrap replicates where this model had the best fit. Similarly clarify this statistic for the other sentences in L430-454.

L480: The meaning of the term "unique loci" is not clear. Does this mean that the 109 loci from study area 1 and the 226 loci from study area 2 did not overlap?

L485: The directionality in RDA refers to the direction of association of a particular *allele* at a SNP, not the SNP itself. The SNP is associated or not; the allele is what increases or decreases in frequency with a particular environmental factor.

L493: The statistical test of variation in SNP densities is not currently described in the Methods section.

L570: Higher diversity compared to what?

L571: In what way were the results similar to those of Arruda et al.? This paragraph is very vague.

L575: What is meant by "moderate" genetic structure?

L585: What are some possible factors influencing individual dispersal patterns? Natal habitat induction / other mechanisms of habitat selection?

L588-589. "The second order river… limited (but not hampered) dispersal and gene flow…" This sentence is vague. How are "limited" and "hampered" being distinguished?

L598-599: "Individual dispersal among these water bodies in search of favorable conditions for shelter, feeding and reproduction would likely be reached at certain density of water bodies." This sentence is unclear. What do the authors mean by "individual dispersal… would likely be reached?"

L606: It is not clear how the observed spatial genetic patterns relate to population dynamics.

L618: It is important to maintain the distinction between gene flow and dispersal, which are frequently conflated in this manuscript. Because the landscape resistance patterns are inferred using pairwise genetic distances, they reflect the impact of landscape features on the effective number of migrants exchanged between locations (i.e., gene flow). The effective number of migrants is a function of both local effective population sizes and migration rate, which are not easily disentangled in the absence of whole-genome data. Thus, landscape resistance patterns might reflect variation in local effective population size rather than behaviors influencing migration rates (e.g., avoidance of open areas). Secondly, gene flow occurs as a result of

dispersal *and successful reproduction*, such that patterns of gene flow can depart markedly from patterns of dispersal.

L651: "… they were more than double in P1O…" What was more than double in P1O?

L660;L681: Genes themselves cannot be directionally associated with environmental variation using these analyses; this wording implies that a gene is or is not present in certain environmental conditions, which is not typically the case. It is more accurate to state that allele/variants at those genes can be directionally associated with environmental variation (e.g., one allele is found at higher frequency in certain environmental conditions).

L632: The identification of unique signatures of selection across the two study areas could reflect statistical limitations rather than real biological differences. For example, correlations between environmental factors and population structure may differ between the two study areas. GEA tests that account for population structure will have the best performance when the environment and population structure are uncorrelated (e.g., see the simulations in Lotterhos et al. 2023). Thus, a candidate SNP might only be detected as having a GEA in the study area where the environment and population structure are least confounded despite truly being under selection in both study areas; this would result in the erroneous conclusion that the SNP is under selection in only one of the study areas (i.e., an erroneous conclusion of non-concordant selection at this locus). The authors could investigate this to some extent by correlating locality genomic PC scores with environmental conditions in each study area and discerning whether the degree of confounding between population structure and the environment differs between the study areas. This possibility should be discussed in a "Limitations" paragraph in the Discussion.

Lotterhos, K. E. (2023). The paradox of adaptive trait clines with nonclinal patterns in the underlying genes. *Proceedings of the National Academy of Sciences*, *120*(12), e2220313120. https://doi.org/10.1073/pnas.2220313120

L628-726: Candidate genes are described at length over approximately four pages. This is an excessive amount of text dedicated to speculating on the functional consequences of variation at candidate genes. I suggest focusing on a few of the most notable candidate genes (e.g., those with the most functional information or similar signatures of selection in other amphibian species). The remaining genes and putative functions can be summarized in a supplementary table.

L728: This manuscript needs a section that explicitly details the limitations of this study. One of the major limitations is the sampling design. The relatively small number of sampling localities (8 for landscape 1; 7 for landscape 2) will tend to limit power to detect genomic signatures of local adaptation. Selmoni et al. (2020) suggest that power to detect GEAs is low when fewer than 20 sampling sites are used (here, this would mean 20 sampling sites per landscape/study area). Furthermore, a total sample size of >200 individuals (i.e., per study area) is recommended for achieving high power in GEA analyses; the per-landscape/study area sample size in this study is

quite small (125 and 65 samples in landscape one and two, respectively) compared to these recommendations. Small numbers of localities and individuals can also inflate the false discovery rate (i.e., the proportion of significant loci that are false positives) well above the nominal level that multiple test corrections are supposed to control it at (Selmoni et al. 2020). The inflation of the false discovery rate can be compensated somewhat by overlapping multiple complementary methods (which the authors do use), at the expense of power (see Forester et al. 2018). This is in no way meant to suggest that the study is not worthy of publication; the authors implemented the GEA tests appropriately, but the inherent limitations of the sampling design need to be acknowledged.

Selmoni, O., Vajana, E., Guillaume, A., Rochat, E., & Joost, S. (2020). Sampling strategy optimization to increase statistical power in landscape genomics: A simulation-based approach. *Molecular Ecology Resources*, *20*(1), 154–169. https://doi.org/10.1111/1755-0998.13095

Forester, B. R., Lasky, J. R., Wagner, H. H., & Urban, D. L. (2018). Comparing methods for detecting multilocus adaptation with multivariate genotype-environment associations. *Molecular Ecology*, *27*(9), 2215–2233. https://doi.org/10.1111/mec.14584

Figure 4: The use of different color gradients across panels makes patterns less interpretable. High resistance and low resistance should always be assigned the same colors.

Figure S4: In panel A, please indicate the proportion of variance explained by each principal component.

Figure S6: Isolation by distance for species inhabiting two-dimensional habitats should be analyzed by regressing linearized FST (i.e., FST/[1 – FST]) against the natural logarithm of geographic distance, as described in detail in Rousset (1997). Here, the authors have inappropriately regressed untransformed FST against untransformed geographic distance.

Rousset, F. (1997). Genetic Differentiation and Estimation of Gene Flow from *F* -Statistics Under Isolation by Distance. *Genetics*, *145*(4), 1219–1228. https://doi.org/10.1093/genetics/145.4.1219

Figure S6: Please make the X- and Y-axis scales identical across panels A and B, so that the patterns in each study area can be compared more easily. An alternative is to make a panel C that plots IBD within each study area in the same figure, with a different color for each study area. Furthermore, please indicate in the figure legend which statistical test was used to obtain the R-squared and p-values.

Figure S9: The label for PCAdapt is truncated to “PCAdap” in both panels.
