# Supplementary material for: Functional connectivity patterns of the Giant Toad Rhinella horribilis in anthropogenically modified landscapes
Source: PLoS One. 2025 Oct 15;20(10):e0319111. doi: 10.1371/journal.pone.0319111 (PMC12527146; doi:10.1371/journal.pone.0319111)
Supplement: Attachment — Submitted filename: Response to Reviewers_R1.pdf [file pone.0319111.s005.pdf]

Response to reviewers

We truly appreciate the time and careful revision made by the reviewers and the Academic Editor, and thank their thoughtful and constructive feedback, which helped us to improve the manuscript throughout.

Academic Editor

Comments to the Author:

The study system is quite interesting, and the RADseq approach has power to detect population structure and possibly also signs of positive selection. The reviewers had mainly issues with analyses, from SNP filtering up to genotype to environment analyses. We acknowledge that it is interesting to aim to screen for loci under selection in two sets of populations, though there is some doubt about how best to do this and if it is achievable with this data. The main critiques by the reviewers are listed below, some of them demand substantial work. We ask you to navigate their comments and take a decision on how best to restructure the paper after the adjusted filtering and new analyses. Note, you dont have to do all they request, a scaled back version dropping the last section (genotype to environment association tests or tests of parallel selection) would still have merit.

We thank the Editor and include here a 'Point-by-point response', where we carefully answer every comment and suggestion made. To facilitate locating the changes we made, they are indicated with track changes in the file named “Revised Manuscript with Track Changes”, and with blue lettering in the responses below in this document. Please note that line numbers in this Response letter refer to the document with track changes.

The main concerns are.

1. How comparable are the RADseq datasets? Recommend reanalyses of population structure of the entire set, see reviewer 2 comments. (if there are issues with the overlap, restrict the analyses to several hundred – thousand best overlapping markers).
2. Better describe the filtering (did you use genotype depth, genotype quality, or allele balance filters?) and consider running analyses with and without the stringest HWE filter (rev 1)
3. There are concerns about the power of the FST approach (reviwers recommend alterntive analytical approaches) and if the relatively low SNP coverage in the two datasets makes it unrealistic to actually detect loci under positive selection.
4. The genotype to environment associations could be influenced (masked or enhanced) by population structure.
5. The raw data have to be released on SRA or equivalent.
6. Scripts detailing analyses should also be available, Github or similar.

Importantly, we followed the Editor’s advice and decided to eliminate the last section of the manuscript regarding the “Detection of outlier SNPs and genomic regions potentially under selection”. The entire manuscript was modified accordingly, namely we removed this topic’s theoretical background from the introduction, the associated methods and results, and the discussion sections. We also modified the title to “Functional connectivity patterns of the giant toad Rhinella horribilis in anthropogenically modified landscapes”.

Thus, the focus of the revised version of the manuscript is directed to the functional connectivity and landscape genetics of *Rhinella horribilis* in our two-landscapes study system. Accordingly, it is based on the aims and objectives of the study without considering the identification and functional annotation of candidate loci. For this revised version we carefully considered and applied the reviewers’ indications in terms of best filtering protocols, reanalyses of genetic structure considering the entire data set (points 1, 2 & 4 above), as well as the suggested new analytical approaches where appropriate (point 3).

We fully comply with the Data Availability policy and confirm that the raw data and the assembled GBS data (vcf file) of the Giant Toad *Rhinella horribilis* analyzed are available in Dryad (point 5).

Importantly, we did not develop any specific code for the analyses performed; we followed the scripts provided within each of the programs used, while describing in detail all the parameters applied within the manuscript (point 6). Finally, the metadata (e.g. sampling locations, data per individual), the methods for all analyses, and the genetic results obtained are available in the main text and in the Supporting Information.

Reviewers' comments:

Reviewer #1:

Soria-Ortiz et al. (PONE-D-25-03029) present a landscape genomics study of the giant toad (Rhinella horribilis) in two study areas in Mexico. A primary goal of the study is to identify signatures of spatially varying selection associated with environmental variation and test whether these signatures are concordant or discordant between the two study areas. The authors find some concordant signatures of selection, evident in the same candidate SNPs identified as associated with the same environmental factors in both study areas. Although this manuscript is within the scope of PLoS One, I have two major concerns that motivated me to recommend rejection of the manuscript in its current form. If revised appropriately, the manuscript would be suitable for publication in PLoS One. These two major concerns are the following:

We deeply thank the reviewer for the thorough review of our manuscript and for the detailed comments and suggestions, which we carefully considered, as we explain below in each point indicated.

To facilitate locating the changes we made, they are indicated with track changes in the file named "Revised Manuscript with Track Changes", and with blue lettering in the responses below in this document. Please note that line numbers in this Response letter refer to the document with track changes.

1) The authors appear to have omitted key genotype and SNP filters routinely applied in population genomics. The authors also implemented a Hardy Weinberg Equilibrium filter that is well-known for distorting inference of population genetic differentiation, which is the focus of approximately half of the manuscript. My line comments explain these issues in greater detail. Correcting potential deficiencies in data filtering could substantially alter the results.

We carefully considered and applied the reviewer's indications in terms of best filtering protocols, reanalyses of genetic structure considering the entire data set, and suggested new analytical approaches where appropriate.

2) I believe that the central question asked in the manuscript – that is, whether genomic signatures of local adaptation are repeated in two different study areas – is not addressed with the sophistication offered by recently developed methods. For example, the genome-wide repeatability analysis of Yeaman et al. (2018) or the genome scan for repeatability at individual loci introduced in Booker et al. (2023; full references provided in the relevant line comment below) were specifically designed to address this question; these methods have been validated using simulated data. The authors did not use either of these methods. Instead, the authors used an ad hoc method with unknown reliability.

Importantly, we followed the Academic Editor's advice of presenting "a scaled back version dropping the last section (genotype to environment association tests or tests of parallel selection)". Therefore, we decided to eliminate the last section of the manuscript regarding the "Detection of outlier SNPs and genomic regions potentially under selection". The entire manuscript was modified accordingly; as a result, although we take meticulous account of all the reviewer's comments made in relation to this topic, we do not refer to or describe associated specific changes in the revised version and neither in this response to reviewers.

We are in debt with the reviewer; we revised all the literature indicated and will incorporate all the suggestions to improve our assessment of the genomic signatures of local adaptation in our study system in a follow up manuscript.

My line comments (below) elaborate on the aforementioned major concerns and indicate more minor feedback throughout the manuscript. Relevant lines (L) are indicated at the start of each comment.

Data availability: I note that although the authors intend to release a VCF file containing individual genotypes, the authors do not indicate a mechanism for publicly releasing their raw sequencing data (i.e., demultiplexed raw sequencing reads). The raw data should be released so that alternative genotyping methods could be used if other researchers wish to use these data. Additionally, the authors do not indicate a mechanism for publicly releasing the scripts used to analyze their data; written descriptions of methods in the main text and/or supporting information are often not sufficient for exactly replicating analyses. I recommend that the authors include their analysis scripts in their Dryad repository and/or in a GitHub repository.
We fully comply with the Data Availability policy and confirm that the raw data and the assembled GBS data (vcf file) of the Giant Toad Rhinella horribilis analyzed are available in Dryad (now clearly indicated in the revised version). Importantly, we did not develop any specific code for the analyses performed; we followed the scripts provided within each of the programs used, while describing in detail all the parameters applied within the manuscript. Finally, the metadata (e.g. sampling locations, data per individual), the methods for all analyses, and the genetic results obtained are available in the main text and in the Supporting Information.

L68-69: The homogenizing effect of gene flow does not imply that alleles experiencing spatially varying selection will be lost. It suggests that locally adaptive genetic differentiation may not evolve because selection may be ineffective compared to gene flow.
We modified the text, now it reads: "Yet the locally adaptive variants may not evolve due to the homogenizing effect of gene flow from neighboring populations"

L75-77: these landscape factors likely do not universally decrease functional connectivity across all species. Revising this to state "In amphibians, some landscape factors often negatively affect…" would temper this claim. However, I am not sure this extended discussion of species-specific environmental effects is warranted in the Introduction.
We modified the sentence as suggested. We believe it is important to present this information so the reader familiarizes with a little background of environmental effects on amphibians.

L123: I find the description of the two study areas as distinct landscapes a bit misleading. The two study areas are separated by 70km but occur on the same landmass. More importantly, the authors have presupposed that the samples collected in the two study areas are genetically distinct units that should be analyzed separately. It is possible that the samples in the two study areas represent distinct genetic clusters; however, this hypothesis is not explicitly tested in the authors' population structure analyses, as they carried out genetic clustering algorithms within each study area separately. Grouping sampling localities for further analysis should be motivated by biology (e.g., genetic clusters), not based on arbitrarily defined geographic units. My suggestion here is to 1) first run genetic clustering algorithms at the total dataset level (i.e., both study areas analyzed collectively); and 2) group localities by high-level genetic clusters for further analyses.
We reviewer is right that we should analyze the complete dataset first. Accordingly, we performed new filtering protocols and analyses considering both landscapes together. These new results show that the two landscapes are indeed clearly genetically differentiated.
We performed further genetic diversity, structure and landscape analyses within each landscape, which allowed us to fulfill our main objectives and to identify landscape elements associated with *R.*

horribilis functional connectivity. We believe all this is best explained and clarified with the final structure of the revised version of the manuscript.
See our responses below at each specific point raised.

L144: Inserting the figure legends throughout the manuscript (and separated from the actual figures) made reviewing the figures challenging. In the future, I recommend that the authors either 1) insert both the figures and their corresponding legends throughout the manuscript; or 2) have all figure legends and figures at the end of the manuscript.
We fully agree! However, we followed Plos One's guidelines that indicate "Figure captions must be inserted in the text of the manuscript, immediately following the paragraph in which the figure is first cited (read order). Do not include captions as part of the figure files themselves or submit them in a separate document." AND "Do not include figures in the main manuscript file. Each figure must be prepared and submitted as an individual file."
In this revised version we inserted both the figure and the figure legend where each is first cited; however, the manuscript was sent back to us by the editorial office, indicating we needed to remove the figures from within the text. Let's hope PlosOne 's formating requirements change at some point.

L151-173: The relationship between the environmental variables and human modification of habitat are not clearly explained. This is surprising given that the title of the manuscript indicates that the genomic signatures of local adaptation are associated with "anthropogenically modified landscapes." The environmental factors that were included (e.g., NDVI, water properties) are expected to vary spatially in the absence of anthropogenic activity, such that the connection between landscape genomic patterns and anthropogenic activity is unclear. The importance of human landscape modification could be more explicitly investigated by including some index of anthropogenic activity (e.g., local percent urban landcover; percent agricultural landcover) in the landscape resistance analyses and genotype-environment association (GEA) analyses. Perhaps the authors can more clearly explain how their chosen environmental factors correlate with anthropogenic activity.
Our study landscapes have experienced a decrease in vegetation cover mainly due to anthropogenic modification for more than 400 years, yielding matrices with patches of different land uses. The selection of environmental variables was based on various factors: they are related to key life history traits of the frog species studied, they constitute specific elements of the landscape matrix of the study areas and at the small scale of our analysis, and they allowed us to build landscape metrics that are associated with landscape modification; namely, as more modified is the landscape there is less vegetation cover (NDVI), more impervious surface area, lower soil humidity, less presence of temporal water bodies. Likewise, ambient temperature, relative humidity, solar radiation and evapotranspiration all change and negatively affect *Rhinella horribilis* the more modified is the landscape (diminished or null vegetation cover, less presence of water bodies, and so on).

In Table S1 we describe each of the environmental variables and explain their biological justification (in which for this revised version we added information for better clarity). Also, in the main text in the Methods section where we state the connectivity hypotheses to be tested with the landscape genetics analysis approach (lines 306-338). Both in Table S1 and the main text we describe in detail (and supported by scientific literature) how the different variables associate both with *R. horribilis* and with our predictions in terms of the modification of the habitat. For example "Although amphibian distribution is in general constrained by humidity [67], *R. horribilis* can be present across open areas and can hence tolerate harsh temperature and radiation conditions [31,32]. Likewise, *R. horribilis* is often found in areas with low vegetation cover [31], but rarely near/at urban cities, while bufonids in general prefer more humid habitats [68,69]. Accordingly, we hypothesized that extremely high temperature, evapotranspiration and solar radiation (i.e., detrimental conditions) will limit *R. horribilis* movement in areas with no vegetation cover".

L151-173: The use of in situ, contemporary measurements of environmental variation is welcome, but it is not clear whether the timeframe of measurement is most relevant to selection. The authors state that sampling was conducted in the rainy season of 2019, so I assume here that environmental measurements were taken in the same time period; the authors should clarify whether this is true. This means that selective pressures that might occur at other time periods may be missed. For example, amphibians are generally prone to desiccation, such that variables like maximum annual temperature could be relevant. Additionally, natural selection is a cumulative process, such that longer-term environmental trends might be relevant but not captured by measurements taken over a short time interval. Additional environmental factors can be sourced from public databases such as WorldClim 2.
As the revised version of the manuscript does not include the evaluation of selection pressures and genotype-environment association, this does not further apply.

L176: The manuscript states that in total, 190 samples were subject to DNA extraction. How many samples were from landscape 1, and how many were from landscape 2? Given that analyses were conducted independently for the two study areas, the per-landscape sample size is the more important than the total sample size.
We are sorry we did not explain this clearly. We added this where appropriate in Methods: "We extracted genomic DNA from 190 *R. horribilis* individual samples (125 for P1O and 65 of P2O)", and in Results: "A total of 190 *R. horribilis* adults were sampled, 125 in landscape P1O and 65 in landscape P2O (Table S4) and all were successfully sequenced."

L180: Why was only 5% of the reference genome used for in silico restriction enzyme digestion? Were the results of this fraction of the genome extrapolated to the whole genome to predict the number of RAD loci that would be obtained?
We did that procedure as an exploratory exercise to identify the potentially best restriction enzymes for our data. Nonetheless, the sequencing laboratory we sent our samples to (University of Wisconsin Biotechnology Center) performed their own analyses for enzyme selection (which agreed with our findings). To avoid confusion, we deleted this sentence in the revised version.

L184: please report the total number of reads generated from sequencing as well as the per-sample sequencing depth.
We include this now "The total number of reads generated from sequencing was 1,187,625,595" where indicated.
We also added "the population-study-P1O and P2O datasets included 4088 and 4190 SNPs, respectively, both with an average coverage of 14x".

L193-203. I note that the authors do not appear to have used genotype depth, genotype quality, or allele balance filters. Genotype depth filtering is distinct from what appears to be a per-SNP mean depth filter (indicated in L198); genotype depth filtering recodes individual genotypes to missing data when the genotypes are supported by too few reads. Typical thresholds include recoding genotypes to missing when their depth is <5x or <10x. A genotype depth filter is particularly important here because ddRAD protocols generally preclude the identification and removal of PCR duplicates, such that effective depth and thus genotype quality may be overestimated (see Andrews et al. 2016 for more discussion). Genotype quality filters will similarly remove low-confidence genotypes; a threshold of GQ < 20 or GQ < 30 is typical. Allele balance (AB) refers to evenness among reads supporting the two different alleles present in heterozygous genotypes. Because the expected value of AB is 0.50, typical AB thresholds include recoding heterozygous genotypes to missing when AB is outside the following range: 0.25 ≤ AB ≤ 0.75. These three filters can be implemented in the R package SNPfiltR (DeRaad et al. 2022; full citation below). See also O' Leary et al. (2018) for greater discussion of these filters. These filters will increase the amount of missing data in the genotype matrix and should therefore be used before removing individuals and/or SNPs based on missing data.

Without any information about sequencing depth, as I commented above, it is unclear whether the dataset may contain many low-quality genotypes that could affect inference of population structure and signatures of selection.

Andrews, K. R., Good, J. M., Miller, M. R., Luikart, G., & Hohenlohe, P. A. (2016). Harnessing the power of RADseq for ecological and evolutionary genomics. Nature Reviews Genetics, 17(2), 81–92. https://doi.org/10.1038/nrg.2015.28
DeRaad, D. A. (2022). snpfiltr: An R package for interactive and reproducible SNP filtering. Molecular Ecology Resources, 22(6), 2443–2453. https://doi.org/10.1111/1755-0998.13618
O'Leary, S. J., Puritz, J. B., Willis, S. C., Hollenbeck, C. M., & Portnoy, D. S. (2018). These aren't the loci you're looking for: Principles of effective SNP filtering for molecular ecologists. Molecular Ecology, 27(16), 3193–3206. https://doi.org/10.1111/mec.14792
We deeply appreciate the reviewer's constructive suggestions and the references provided. Taking these filters and those in subsequent comments into account, we performed new post-filters to obtain new SNP datasets. Additionally, given that the revised manuscript does not include the evaluation of selection pressures and genotype-environment association, these datasets we built were for analyzing neutral patterns.
Thus, we built three datasets, one that included all the samples from the two landscapes (study-wide dataset) and two more by landscape (population-study-P1O and population-study-P2O). Additional quality filtering (from that of the raw data filtering process) was done for each dataset. We briefly describe the filtering protocols of these datasets (which include the suggestions of the reviewers) in the main text and provide a detailed description of the filtering processes and results in a new supplementary document (Supplementary Methods). The full description is in the Methods section lines 218-241.

L193-203: I also note that SNPs do not appear to have been filtered for linkage disequilibrium (LD). This is most pertinent to the more stringently filtered dataset intended for detecting population structure. Many population genetic clustering algorithms assume that loci are independent, which may be violated here. I recommend determining the physical extent of substantially elevated LD and thinning SNPs based on that interval. E.g., if LD is substantially higher than background until a distance of 100Kb, the dataset should be thinned to retain one SNP per 100Kb; this can be implemented in VCFtools using the thin command. The larger dataset used for detecting signatures of selection does not need to be thinned for LD because statistics from multiple nearby SNPs can be used to boost the signal of selection (e.g., using the weighted Z analysis or WZA; Booker et al. 2024).

Booker, T. R., Yeaman, S., Whiting, J. R., & Whitlock, M. C. (2024). The WZA : A window-based method for characterizing genotype–environment associations. Molecular Ecology Resources, 24(2), e13768. https://doi.org/10.1111/1755-0998.13768
In this revised version we performed appropriate linkage disequilibrium (LD) filtering. The full explanation is: "We performed two processes for selecting linkage disequilibrium (LD) and minor allele count (mac) filters. First, to decide the cutoff for LD, we started with a filtered study-wide dataset that included: allele balance (0.25-0.75); minDP=5; minQG=20; only biallelic loci; maximum missing data allowed 20% (--*max-missing*); maximum heterozygosity 0.5 (--*max-obs-het*); 2(x) mode depth=34; and minor allele count=3 (--mac). Based on this dataset (54,194 SNPs) we used plink1.9 and ran --r2 to obtain the allele count square root correlation (Hemstrong et al., 2024), using the parameters --ld-window 1000, --ld-window-kb 50, --ld-window-r2 0. Based on the LD decay plot obtained (Figure 1), we chose a string cut-off value of 10kb for the LD filter, for which we found most of the loci with an r2 value below 0.2.
The same was done for the two population-study datasets, also obtaining a string cut-off value of 10kb.
All is explained as indicated above, in the main text and the Supplementary Methods document.

L193-203: I also note that the authors do not appear to have implemented filters that seek to remove SNPs that are at high risk of representing collapsed paralogous sequences. There are two commonly used filters to do this: 1) a per-SNP observed heterozygosity filter (not the same as the HWE filter used by the authors); and 2) a per-SNP mean sequencing depth filter. Point #1 involves removing SNPs with unusually high observed heterozygosity in the total dataset (i.e., among all individuals from all localities). Typically, SNPs with observed heterozygosity > 0.50 are removed. This is because the maximum expected heterozygosity for a biallelic SNP in a panmictic population is 0.50 (occurring when the two alleles are equally frequent); the existence of population structure would make observed values > 0.50 even more unusual. SNPs that have a high observed heterozygosity in the total dataset are likely to represent collapsed paralogous sequences, which should be removed. For Point #2, SNPs with unusually high mean sequencing depth across individuals may also represent collapsed paralogous sequences. Per-SNP mean sequencing depth can be output using the command --site-mean-depth in VCFtools; one rule of thumb is to remove SNPs that have a mean sequencing depth greater than two times the modal value (Willis et al. 2017).

O'Leary, S. J., Puritz, J. B., Willis, S. C., Hollenbeck, C. M., & Portnoy, D. S. (2018). These aren't the loci you're looking for: Principles of effective SNP filtering for molecular ecologists. Molecular Ecology, 27(16), 3193–3206. https://doi.org/10.1111/mec.14792.
Willis, S. C., Hollenbeck, C. M., Puritz, J. B., Gold, J. R., & Portnoy, D. S. (2017). Haplotyping RAD loci: An efficient method to filter paralogs and account for physical linkage. Molecular Ecology Resources, 17(5), 955–965. https://doi.org/10.1111/1755-0998.12647
In this revised version we performed the indicated filters. We removed potential paralogous sequences using a cut-off value of 2x mode, where "x" is per-SNP mean sequencing depth (Willis et al. 2017), and SNPs with observed heterozygosity >0.05 were removed.

L194: "each one" meaning each landscape? Please clarify.
Writing of this entire section was modified, now it reads (226-229): "From this first SNPs dataset and considering the objectives of our study, we built three datasets, one that included all the samples from the two landscapes (study-wide dataset) and two more by landscape (population-study-P1O and population-study-P2O). Additional quality filtering was done for each dataset …"

L198: It is not clear what "minimum depth of 10 samples to recognize a SNP as true" means. Does this mean that the mean depth across samples for a given SNP had to be ≥ 10 reads? Additionally, please replace "recognize a SNP as true" to "retain a SNP," which makes no claim about whether the SNP is likely a real polymorphism.
Writing of this entire section was modified, now it says (lines 232-238): "… we assessed allele balance using *filter_allele_balance* with the default parameters [46]; applied genotype depth and genotype quality using --minDP=5 and --minGQ=20, respectively [47]; only biallelic loci were retained; maximum allowed amount of missing data of 20% (--*max-missing*); and a string cut-off value of 10kb for the linkage disequilibrium (LD) filter (--*thin*) (see specific details in Supplementary Methods). We also removed potential paralogous sequences using a cut-off value of 2x mode, where "x" is per-SNP mean sequencing depth [48], and SNPs with observed heterozygosity >0.50 (--*max-obs-het*). "

L199-203: MAF is the abbreviation for minor allele frequency, not minimum allele frequency. The MAF threshold of 0.05 for tests for selection is consistent with best practices highlighted in Ahrens et al. (2021) and is fine. However, the MAF threshold of 0.10 for population structure analyses runs counter to best practices identified in the literature, where SNPs with rare but not singleton alleles should be kept to improve assignment accuracy (e.g., Linck and Battey 2019). A minor allele count threshold of 3 retains rare but not singleton alleles; this can be implemented in VCFtools using the command - -mac 3.

Linck, E., & Battey, C. J. (2019). Minor allele frequency thresholds strongly affect population structure inference with genomic data sets. Molecular Ecology Resources, 19(3), 639–647. https://doi.org/10.1111/1755-0998.12995

Thank you for noticing the mistake. Given the neutral datasets we use in the study, we only applied mac filters. Lines 239-241: "Finally, we assessed a range of minor allele count (--*mac*) values (3 to 17; see Supplementary methods S1 for all the results), and chose a minor allele count of seven for both the study-wide and the population-study-P1O datasets and of five for the population-study-P2O."

L200-201: The use of a HWE filter is problematic because population structure creates deviations from HWE (the Wahlund effect). Removing loci showing strong deviations from HWE can distort estimates of population genetic differentiation. See Pearman et al. (2022; full citation below).

Pearman, W. S., Urban, L., & Alexander, A. (2022). Commonly used Hardy–Weinberg equilibrium filtering schemes impact population structure inferences using RADseq data. Molecular Ecology Resources, 22(7), 2599–2613. https://doi.org/10.1111/1755-0998.13646

We thank the reviewer for highlighting this; accordingly, we eliminated the HWE filtering.

L205-226: I note that all of the genetic clustering methods used by the authors assume discrete genetic groups. Even TESS, which incorporates spatial information, assumes that allele frequencies are spatially homogenous within each genetic cluster. It is clear from Figure S6 that both study areas show a strong pattern of isolation by distance (IBD), suggesting that this species is better characterized as a spatially continuous population rather than a set of discrete genetic groups. A consequence of IBD is that discrete clustering algorithms will tend to overestimate the number of genetic groups in the data (e.g., see Fig 2 in Bradburd et al. 2018; full citation below). I recommend implementing the analysis ConStruct (Bradburd et al. 2018), which implements both a non-spatial, discrete genetic clustering model (similar to ADMIXTURE) and a spatially explicit genetic clustering model. Essentially, the spatial model explains observed genetic variation as reflecting the contributions of multiple "geographic-genetic layers" that are interpreted similarly to genetic clusters in discrete algorithms. However, in contrast to non-spatial discrete models, the spatial model accommodates greater biological realism by allowing genetic similarity to decay with geographic distance within each geographic-genetic layer. ConStruct also provides a way to explicitly compare the predictive performance of its non-spatial and spatial models (i.e., model selection). Given that the authors are studying spatial patterns, a spatially explicit analysis such as ConStruct would perhaps yield inferences of population structure that better reflect reality.

Bradburd, G. S., Coop, G. M., & Ralph, P. L. (2018). Inferring Continuous and Discrete Population Genetic Structure Across Space. Genetics, 210, 33–52.

Our thanks for the advice and suggestions.
It is important to note that with the filtering additions/changes and the new SNPs datasets generated, the results for the complete dataset demonstrated that the two landscapes are indeed genetically differentiated.
While for each landscape, structuring results (based on SNMF, TESS, DAPC, PCA, sPCA) improved, showing more clearly *K*=3 and *K*=2, for landscape P1O and P2O, respectively.

As suggested by the reviewer, we implemented this continuous structure method for each landscape following Bradburd's protocol (https://github.com/construct/construct): "To account for the potential presence of isolation by distance in landscapes, we use ConStruct (Bradburd et al., 2018). This method allows for explicit test of discrete versus continuous geographic differentiation, estimating the rate at which allele frequency relatedness decays with distance. Spatial and non-spatial models were tested with *K* ranging from 1 to 10, and 10 repetitions, using 20000 iterations. Cross-validation and layer contribution were performed to determine the most likely number of clusters (best *K*)."

The results obtained did not provide a better resolution for the genetic structuring patterns associated with our data. Based on cross-validation for the selection of *K* in the spatial model, the best *K* value was between 2 and 3 for both landscapes. We include a pdf document (ConStruct_Analysis_R1) with explanations and figures of the *K*=2, *K*=3 plots for each landscape as evidence but, because the results do not show a structure that better reflected reality and they do not make spatial sense, we chose not to include the ConStruct analyses in the manuscript.

L207: I would like to hear from the authors whether there might be some value to identifying population genetic clusters and describing population genetic differentiation at the total dataset level (i.e., by running analyses using all individuals from the two study areas collectively). Currently, the manuscript presupposes that the two study areas are genetically distinct units that should be analyzed separately. However, they occur in the same landmass just 70km apart, and weak differentiation within each study area (max FST ~ 0.03) suggests that geographic boundaries between genetic clusters may not align with the study areas (e.g., some localities in landscape 2 might actually assign to genetic clusters in landscape 1 or vice versa), in which case the dataset should be subdivided by genetic cluster rather than arbitrarily defined landscape areas. Including population structure analyses at the total dataset level might also give some context to the identification of repeated signatures of genetic-environment associations. E.g., if there is only weak genetic differentiation between the two study areas, it may be that the genetic clusters in the two study areas have adapted to repeated environmental gradients from shared standing variation.
We thank the reviewer for highlighting this. As explained in the answers above, we built a dataset with all the 190 samples (study-wide dataset) and performed (in addition to all the filtering) structure analyses using PCA and SNMF, which clearly identified two distinct genetic groups (Fig 1d,e), namely P1O and P2O are genetically differentiated.

L214: The authors have applied DAPC using groups of samples defined a priori (i.e., the sampling localities). It is also possible to use K-means clustering to objectively define genetic clusters based on the genetic data and follow that with DAPC; i.e., using the function find.clusters and following it with the typical DAPC workflow. I bring attention to this because it is not clear what the purpose of DAPC here is; if it is to cluster samples genetically, then the objective K-means workflow should be used.
We appreciate the observation. DAPC uses the *a*-score method to determine the proportion of successful reassignment by individual as a function of the number of retained PCs, which maximizes the differentiation between populations, which we aimed to do when analyzing structure within each landscape (population level).

We added in the revised version the explanation (lines 263-267) that "Miller et al. [51] showed that using *a priory* grouping for DAPC is more efficient to better reflect the underlying F_{ST}, even if it is small, and when sampling sizes are small, as it is the case in the two landscapes studied. Therefore, we grouped individuals *a priory* according to the eight and seven sampling sites in P1O and P2O, respectively and ran DAPC with *dapc* in adegenet."

Importantly, DAPC results were in agreement with those of the other structuring methods used, supporting that three well defined genetic groups were identified in P1O and two in P2O.

This is clearer now in the revised version with the changes made and improved explanations in the Methods and Results.

Miller, J. M., Cullingham, C. I., & Peery, R. M. (2020). The influence of a priori grouping on inference of genetic clusters: simulation study and literature review of the DAPC method. Heredity, 125(5), 269-280.

As the revised version of the manuscript does not include the evaluation of selection pressures and genotype-environment association, from here (L328) down to the comment L357-375: they do not further apply.

L328: Which correlation coefficient was used (E.g., Pearson's, Spearman's, Kendall's)?

L329: I note that lfmm_ridge is one implementation of LFMM 2, not the original LFMM that is cited here as reference 74. The appropriate reference for LFMM 2 is Caye et al. (2019; full citation below). Caye, K., Jumentier, B., Lepeule, J., & François, O. (2019). LFMM 2: Fast and Accurate Inference of Gene-Environment Associations in Genome-Wide Studies. Molecular Biology and Evolution, 36(4), 852–860. https://doi.org/10.1093/molbev/msz008

L324: RDA models can be used to evaluate variance inflation factors (VIFs) and remove highly correlated environmental factors. This process is different from evaluating pairwise correlations between environmental factors (which the authors have done, as noted in L319) because VIFs assess how multicollinearity among predictors leads to instability in model parameter estimation.

I recommend that the authors use a VIF-based environmental factor thinning procedure for their RDA (e.g., remove environmental factors with VIF > 10).

L326: How many permutations were used for anova.cca?

L333: The authors state that they "… ran these analyses with K=3." Which analyses are being referred to? I note that earlier in this paragraph, the authors describe RDA, which does not account for population structure; if the authors accounted for population structure in RDA, they should describe the use of a partial RDA (pRDA) procedure.

L334: The use of a multiple test correction and choice of significance threshold are not indicated for LFMM.

L338: How were candidate SNPs assigned to genes? Proximity?

L357-376: It is not clear what the authors mean by testing one study area's candidate SNPs "in the other landscape." Figure S3 does not clarify this.

L376: There is a lack of detail regarding the use of Fisher's exact test. E.g., were multiple tests done for different environmental factors?

L357-375: I note that there are specialized methods for detecting repeated signatures of selection among multiple lineages, none of which are used in this manuscript. E.g., the C-score method of Yeaman et al. (2018) takes as input the test statistic associated with every SNP for two or more lineages and determines whether the genome-wide test statistic values are more similar between the lineages than expected by chance. This could be done multiple times for each environmental factor. To run this analysis, the authors will have to subset the SNPs to include only those present in both study areas. Alternatively, the authors could first aggregate per-SNP statistics into genomic windows using a method like the weighted Z analysis (WZA; Booker et al. 2024) so that SNPs only identified in one study area can still contribute to inference; the C-score analysis can then be run on genomic window-level statistics. I note also that Booker et al. (2023) recently introduced a formal genome scan for repeated evolution; this method can identify signatures of repeated evolution at individual loci rather than the genome-wide inference provided by the C-score analysis. Both methods are relevant here. Full citations for these references are given below.

Yeaman, S., Gerstein, A. C., Hodgins, K. A., & Whitlock, M. C. (2018). Quantifying how constraints limit the diversity of viable routes to adaptation. PLOS Genetics, 14(10), e1007717. https://doi.org/10.1371/journal.pgen.1007717

Booker, T. R., Yeaman, S., Whiting, J. R., & Whitlock, M. C. (2024). The WZA : A window-based method for characterizing genotype–environment associations. Molecular Ecology Resources, 24(2), e13768. https://doi.org/10.1111/1755-0998.13768

Booker, T. R., Yeaman, S., & Whitlock, M. C. (2023). Using genome scans to identify genes used repeatedly for adaptation. Evolution, 77(3), 801–811. https://doi.org/10.1093/evolut/qpac063

L379: I note that the SNP dataset has 190 total samples, which was the number of individuals that were initially sequenced. L200 states that individuals with >20% missing data were removed. In practice, were any individuals removed? I.e., are the 125 landscape 1 and 65 landscape 2 individuals the final, post-filtering sample sizes for the two landscapes?
Thanks for the observation. The 20% we mentioned actually refers to missing data for genotypes, not individuals, which is why the sample number was not affected. We corrected the writing in the Methods (as shown in our answers above)

L379: I commented on this earlier, but the per-landscape sample sizes should be indicated in the Methods near L176 where the total sample size is first reported. The per-landscape sample sizes are most important given that analyses were conducted independently in the two landscapes/study areas.
Done. "We extracted genomic DNA from 190 *R. horribilis* individual samples (125 for P1O and 65 of P2O)", and in Results: "A total of 190 *R. horribilis* adults were sampled, 125 in landscape P1O and 65 in landscape P2O (Table S4) and all were successfully sequenced."

L380: The reduction from ~4.6M "raw" SNPs to ~10K is not unusual for RAD data, but it would be informative to have a supplementary table that indicates the number of SNPs retained after each filtering step.
We are sorry for the confusion; we had an error that now is corrected. We initially started with "1,134,057 polymorphic sites", then we conducted post-filtering processes (in Methods and some of our answers above). Indeed, as suggested we now summarized the results in a supplementary table (in Supplementary Methods Tables 1 and 2) indicating the number of SNPs retained after each filtering step.

L380-382: Calling the more stringently filtered dataset "neutral data" is potentially misleading. It would be clearer to call this the "more stringently filtered dataset used for population structure analyses." Similarly, the less stringently filtered dataset ("full data") would more clearly be described as the dataset used for GEA analyses. I also note that the authors do not describe removing candidate SNPs from the "neutral" dataset, which further calls into question this terminology.
This was completely modified both because the GEA analyses (and the dataset used for it) were eliminated from revised version of the manuscript, and because as explained we built new stringent datasets. A study-wide dataset for cross-landscape analysis, and population-study datasets for each landscape separately.

L381-384: "Average coverage" is not meaningful. Is this referring to the mean across SNPs, across individuals, or both?
This line was deleted.

L384: What were the final statistics regarding missing data? I.e., mean and range of missing data per SNP, mean and range of missing data per individual?
The missing data was at the genotype level. We corrected it

L387: The Methods section does not describe the statistical test for comparing genetic diversity between the two study areas. It looks like a t-test may have been used, which should be described in the Methods. Importantly, the authors should confirm whether the assumptions of the t-test were met; this process is also not described. Additionally, what was the unit of observation – locality?
We are sorry for the confusion. It is now indicated as (lines 281-286): "Given that P1O and P2O are learly genetically separated (see Results), genetic diversity, measured as observed (H_o) and expected (H_e) heterozygosity, as well as F_{IS}, were estimated per landscape with *populations* in Stacks; statistical differences between landscapes were evaluated using a t-test in R." Yes, the unit of observation was sampling locality.

L401-403: This sentence strikes me as interpretation, which should not occur in the Results section.
We do not agree; we consider this is a necessary precision within the context of the structuring results being described.

L423: why do the R-squared estimates and p-values indicated here differ from those shown in Figure S6?
We are sorry! it was a typo. They now match with those in the (new) corresponding figures.

L430-454: Please include the "%" symbol after each R-squared value.
Done

L431: Please indicate that "85.6%" refers to the percent of pseudo-bootstrap replicates where this model had the best fit. Similarly clarify this statistic for the other sentences in L430-454.
To avoid having too much repetitive text along the manuscript, we indicated this in each Table 1 and Table 3: "Best-supported models are indicated by highest 'top model (%; refers to the percent of pseudo-bootstrap replicates where this model had the best fit)' in bold"

As the revised version of the manuscript does not include the evaluation of selection pressures and genotype-environment association, from here (L480) down to the comment L493:
they do not further apply.
L480: The meaning of the term "unique loci" is not clear. Does this mean that the 109 loci from study area 1 and the 226 loci from study area 2 did not overlap?
L485: The directionality in RDA refers to the direction of association of a particular allele at a SNP, not the SNP itself. The SNP is associated or not; the allele is what increases or decreases in frequency with a particular environmental factor.
L493: The statistical test of variation in SNP densities is not currently described in the Methods section.

L570: Higher diversity compared to what?
We changed the wording for clarity: "However, compared to those studies [19,82-84], we observed overall similar genetic diversity in the *R. horribilis* populations studied"

L571: In what way were the results similar to those of Arruda et al.? This paragraph is very vague.
We are sorry for the confusion. We changed the wording for clarity: "Similar to our findings, Arruda and collaborators [85] report moderate genetic diversity and low inbreeding (measured with microsatellite loci) in populations of the Cururu Toad *Rhinella schneideri* inhabiting agricultural areas. Such patterns may be related with certain life history traits of the two species, …"

L575: What is meant by "moderate" genetic structure?
We eliminated the sentence. It now reads, "Notably, we found as expected lower genetic diversity, higher genetic structure and lower functional connectivity among populations in the landscape with higher habitat modification (P1O), also exhibiting isolation by barrier associated with the main river and roads (the latter in P2O as well)."

L585: What are some possible factors influencing individual dispersal patterns? Natal habitat induction / other mechanisms of habitat selection?
Great suggestion, we added the following (lines 592-595): "For instance, individual dispersal at these fine scales can be associated with sites that provide favorable conditions for shelter, feeding and

reproduction [85-87], like the temporary water bodies in our study sites distributed in pasture lands near patches of high cover vegetation."

L588-589. "The second order river… limited (but not hampered) dispersal and gene flow…" This sentence is vague. How are "limited" and "hampered" being distinguished?
We eliminated (but not hampered)

L598-599: "Individual dispersal among these water bodies in search of favorable conditions for shelter, feeding and reproduction would likely be reached at certain density of water bodies." This sentence is unclear. What do the authors mean by "individual dispersal… would likely be reached?"
It was indeed confusing; we eliminated the sentence.

L606: It is not clear how the observed spatial genetic patterns relate to population dynamics.
We rephrased the sentence as: "Therefore, it is crucial to conserve these landscape features to maintain populations of this and other anuran species."

L618: It is important to maintain the distinction between gene flow and dispersal, which are frequently conflated in this manuscript. Because the landscape resistance patterns are inferred using pairwise genetic distances, they reflect the impact of landscape features on the effective number of migrants exchanged between locations (i.e., gene flow). The effective number of migrants is a function of both local effective population sizes and migration rate, which are not easily disentangled in the absence of whole-genome data. Thus, landscape resistance patterns might reflect variation in local effective population size rather than behaviors influencing migration rates (e.g., avoidance of open areas). Secondly, gene flow occurs as a result of dispersal and successful reproduction, such that patterns of gene flow can depart markedly from patterns of dispersal.
We deleted "that is, the toads seem to avoid open areas with high temperature and UV exposure"

As the revised version of the manuscript does not include the evaluation of selection pressures and genotype-environment association, from here (L651) down to the comment L738:
they do not further apply.
L651: "… they were more than double in P1O…" What was more than double in P1O?
L660;L681: Genes themselves cannot be directionally associated with environmental variation using these analyses; this wording implies that a gene is or is not present in certain environmental conditions, which is not typically the case. It is more accurate to state that allele/variants at those genes can be directionally associated with environmental variation (e.g., one allele is found at higher frequency in certain environmental conditions).
L632: The identification of unique signatures of selection across the two study areas could reflect statistical limitations rather than real biological differences. For example, correlations between environmental factors and population structure may differ between the two study areas. GEA tests that account for population structure will have the best performance when the environment and population structure are uncorrelated (e.g., see the simulations in Lotterhos et al. 2023). Thus, a candidate SNP might only be detected as having a GEA in the study area where the environment and population structure are least confounded despite truly being under selection in both study areas; this would result in the erroneous conclusion that the SNP is under selection in only one of the study areas (i.e., an erroneous conclusion of non-concordant selection at this locus). The authors could investigate this to some extent by correlating locality genomic PC scores with environmental conditions in each study area and discerning whether the degree of confounding between population structure and the environment differs between the study areas. This possibility should be discussed in a "Limitations" paragraph in the Discussion.

Lotterhos, K. E. (2023). The paradox of adaptive trait clines with nonclinal patterns in the underlying genes. Proceedings of the National Academy of Sciences, 120(12), e2220313120. https://doi.org/10.1073/pnas.2220313120
L628-726: Candidate genes are described at length over approximately four pages. This is an excessive amount of text dedicated to speculating on the functional consequences of variation at candidate genes. I suggest focusing on a few of the most notable candidate genes (e.g., those with the most functional information or similar signatures of selection in other amphibian species). The remaining genes and putative functions can be summarized in a supplementary table.
L728: This manuscript needs a section that explicitly details the limitations of this study. One of the major limitations is the sampling design. The relatively small number of sampling localities (8 for landscape 1; 7 for landscape 2) will tend to limit power to detect genomic signatures of local adaptation. Selmoni et al. (2020) suggest that power to detect GEAs is low when fewer than 20 sampling sites are used (here, this would mean 20 sampling sites per landscape/study area). Furthermore, a total sample size of >200 individuals (i.e., per study area) is recommended for achieving high power in GEA analyses; the per-landscape/study area sample size in this study is.

Reviewer #2:
Soria-Ortiz et al. used RADseq in a large sample (n = 190) of Rhinella horribilis from two geographic regions in Oaxaca, Mexico, to generate the first population genomic characterization of this species. The two geographic regions that the authors focused on differed substantially in their degree of anthropogenic land transformation, which enabled the authors to make inferences about how human-driven environmental change affects genome-wide diversity and genetic connectivity. Furthermore, the authors used multiple approaches to identify functional loci likely involved in adaptation to the different environments, highlighting the NOTCH and MAPK pathways as likely being key to adaptation to anthropogenetically modified habitats in R. horribilis, and possibly other amphibians. I think that this is extremely valuable and insightful work from both an evolutionary perspective, especially concerning the genetic basis of rapid adaptation to human-modified environments, as well as from a conservation perspective. I generally thought that the author's analyses displayed ingenuity and were sound. I also enjoyed the discussion of the potential functional roles of the candidate adaptive loci within the different environmental contexts presented in the Discussion; I often feel that such discussions are quite speculative, but in this case seemed well-supported and sensible, especially given the other cited evidence. Below, I provide some ways in which I think the authors can improve this work to ensure the accuracy of their results and inferences.
We truly appreciate the enthusiastic comments of the reviewer, and thank the time and careful revision made, as well as the constructive feedback that helped us to improve the manuscript throughout.

Importantly, we followed the Editor's advice and decided to eliminate the last section of the manuscript regarding the "Detection of outlier SNPs and genomic regions potentially under selection". The entire manuscript was modified accordingly, namely we removed this topic's theoretical background from the introduction, the associated methods and results, and the discussion sections. We also modified the title to "Functional connectivity patterns of the giant toad *Rhinella horribilis* in anthropogenically modified landscapes".
Thus, the focus of the revised version of the manuscript is directed to the functional connectivity and landscape genetics of *Rhinella horribilis* in our two-landscapes study system. Accordingly, it is based on the aims and objectives of the study without considering the identification and functional annotation of candidate loci.

We are in debt with the reviewer for the comments regarding the removed section; we will incorporate all the suggestions to improve our assessment of the genomic signatures of local adaptation in our study system in a follow up manuscript.

(1) My first main concern relates to linking landscape features with genetic connectivity, specifically, using FST as a measure of (especially recent) gene flow. FST is a fixation index, and while it is informative about allelic differences between populations, which is affected by gene flow, FST is also affected by other demographic factors, like the effective size of populations. Furthermore, FST reflects long-term average migration rates and so while it is likely sensitive to older geographic features like rivers, FST may not be very sensitive to more recent landscape barriers or conduits to gene flow like roads, agriculture, etc., which seems important to this study. I am not saying that it is wrong to use FST to make inferences about connectivity, even on recent timescales, but it is important to consider how pairwise FST is affected by the demography, specifically, the rate of genetic drift, within the populations being compared to isolate the effects of environment on migration specifically, and also about whether FST is even sensitive enough to detect changes in migration rates arising from recent habitat modification. My suggestions are, in addition to FST as a proxy for gene flow, to examine rates of rare variant sharing between populations and apply

methods that directly estimate migration rates. For example, you could consider the proportion of shared doubletons between subpopulations, that is, doubletons that are characterized by one copy of the globally rare allele occurring in one subpopulation and the other copy occurring in the other subpopulation (versus both copies of the rare allele within one of the subpopulations). Rare allele sharing of this kind would be consistent with recent migration because most globally rare variants would be relatively young and so the most likely way they are shared between populations is from recent migration. I suspect that looking at rare variant sharing would be more powerful for detecting differences in connectivity at more recent timescales. In addition, I would strongly suggest trying to use an approach like FEEMS (https://doi.org/10.7554/eLife.61927) to make direct inferences about migration. The migration surfaces produced by FEEMS would be ideal for associating with the landscape feature surfaces that the authors have generated. Also, since the authors focus on functional connectivity it could be useful to compare genome-wide estimates of genetic connectivity to estimates of gene flow at candidate adaptive loci in relation to different landscape features or environmental gradients.

We appreciate the observation. Accordingly, we used a second genetic distance metric, the 'proportion of shared alleles' (*Dps*; estimated as as 1-Dps). This metric has been amply used in landscape genetics; it is more sensitive to detecting recent migration signals (Murphy et al., 2010). We also chose to continue working with *Fst*, and performed all genetic landscape analyses with both distance metrics.

We explain this in lines 296-301: "We estimated two genetic distances, F_{ST} and the inverse of the proportion of shared alleles (*Dps*) between sampling sites within each landscape, using *pairwise.neifst* with hierfstat and *pairwise.propShared* with adegenet, respectively. F_{ST} is a measure of population differentiation due to genetic structure, while *Dps* is more sensitive to recent demographic events [57,58]. Given that F_{ST} is widely used in genetic studies, we include it as a metric that can be compared with other studies [59]."

Murphy, M. A., Evans, J. S., & Storfer, A. (2010). Quantifying *Bufo boreas* connectivity in Yellowstone National Park with landscape genetics. Ecology, 91(1), 252-261.

Importantly, the results of the multivariate model selection showed for both landscapes (P1O and P2O) and both genetic distances (F_{ST} and *Dps*) that the Aquatic model (Temporary water bodies + Temporary streams) was the best-supported model and the one with highest genetic variation explained.

Regarding the suggestion to use FEEMS, which we appreciate, we chose not to perform it for two reasons; one is that it is not the best fit for the kind of data we have (i.e. sparse sampling; Marcus et al. 2021); also because we tested a spatially continuos method (ConStruct) to account for the potential presence of isolation by distance within landscapes. The results obtained did not provide a better resolution for the genetic structuring patterns associated with our data (not shown in the revised version).

(2) A second issue that I was left wondering about is how much differences in SNP sets, the distribution of linkage disequilibrium across the genomes, and differences in LD between the P10 and P20 affect inferences about the similarity/dissimilarity of the genomic architecture of environmental adaptation between the P10 and P20 toads. The density of quality controlled SNPs, even in the more liberally filtered set of SNPs, was low given the R. marina genome size (only 8,753 and 13,544 SNPs in the P10 and P20 groups, respectively), and surely adaptive signals from any truly causal adaptive variant is being detected through linkage. For interpreting overlap and differences in the genetic underpinnings of adaptation to different environments and identifying which environmental factors are relevant given the analyses that the authors performed between P10 and P20 toads, I would argue that it is important to analyze the same set of genomic sites in both groups, otherwise the

discovery of candidate loci between groups is biased. In addition, because adaptive loci are likely to be detected through linkage of analyzed SNPs to casual variants (and not from the causal variants themselves) it will be important to know how the distribution of LD differs between P10 and P20 groups, so I would suggest calculating pairwise LD between SNPs within the P10 and P20 groups using Plink or ngsLD, for example, noting that population structure will influence estimates of LD and so calculations might need to be carried out within subpopulations if structure is strong. Given a common set of SNPs between groups, if LD is higher in one group due to, e.g., smaller Ne, one could be more likely to discover more candidate adaptive loci in a RAD dataset. I realize that interesting loci might be lost by paring down to a common set of quality sites shared between P10 and P20 groups, however for comparative analyses I think this is important. The authors can still present all potentially adaptive loci discovered within each of the groups given their full respective sets of SNPs (as is done now), but I think the authors should approach comparing sets of SNPs and loci between groups with caution and mindfulness of the biases that using different SNP sets involves.

I am also not sure whether "loci", for example in lines 478–482, is being used synonymous with "SNP". That is, do the authors mean a SNP, a region, a gene, etc? It would be helpful to readers if the authors were specific about what a locus refers to throughout the paper.
As the revised version of the manuscript does not include the evaluation of selection pressures and genotype-environment association, the two comments above not further apply.

In general, all inferences about neutral genetic processes (estimates of diversity, FST, migration, genome-wide structure, etc) should be based on the same set of sites for both the P10 and P20 groups. Likewise, at least for inferences involving comparisons between the P10 and P20 groups, the 'full data' set should consist of the same sites for P10 and P20. Otherwise, comparing estimates of population genetic parameters, summary statistics, and sets of identified adaptive loci will be biased.
The reviewer is right and we thank for highlighting it.
Indeed, we made important changes regarding the datasets used and the filtering protocols.
First, given that the revised manuscript does not include the evaluation of selection pressures and genotype-environment association, the datasets we built were for analyzing neutral patterns.

Thus, we built three datasets, one that included all the samples from the two landscapes (study-wide dataset) and two more by landscape (population-study-P1O and population-study-P2O). Additional quality filtering (from that of the raw data filtering process) was done for each dataset. We briefly describe the filtering protocols of these datasets (which include the suggestions of the reviewers) in the main text and provide a detailed description of the filtering processes and results in a new supplementary document (Supplementary Methods).

We performed analyses considering both landscapes together. These new results show that the two landscapes are indeed clearly genetically differentiated.
We performed further genetic diversity, structure and landscape analyses within each landscape, which allowed us to fulfill our main objectives and to identify landscape elements associated with *R. horribilis* functional connectivity. We believe all this is best explained and clarified with the final structure of the revised version of the manuscript.

(3) My third major recommendation relates to vetting the candidate loci and to ruling out the potential of false positives (and making inferences based on them), and to generally boost confidence in the validity of the discovered candidate loci. Given that candidate loci were chosen based on statistical tests, I would recommend that the authors present Q-Q plots of the expected versus observed SNP p-values for the various environmental association tests. Providing such Q-Q plots in the supplement will help the authors and other researchers be confident that the statistical

tests behaved as they should, and were not being confounded by population structure for example, which would tend to inflate signals of association across the genome and lead to the discovery of falsely significant loci. It was unclear to me whether confounding ancestry/population structure was being sufficiently accounted for in the genetic association tests with environment; presenting Q-Q plots would make things like this clear. I appreciated that the authors tested how much environmental variation was explained by randomly chosen subsets of SNPs versus the candidate loci, which does strongly suggest that population structure is not a confounding factor in the association testing, but it would still be nice to see the p-value Q-Q plots.

Also, assuming that there is sufficient density of high quality SNPs across the genome (which could likely be achieved through modified SNP filtering), I recommend that the authors test whether patterns of genetic diversity are consistent with selection acting on the candidate adaptive loci, which will help validate the candidates. The authors could likely use R. marina as an outgroup to perform HKA tests for non-neutral evolution at the candidate loci and identify what types of selection are responsible for their adaptive evolution. With sufficient SNP density, the authors could also use statistics like Tajima's D or other local SFS-based approaches (e.g., SweepFinder) to assess whether the distribution of genetic diversity at candidate loci is consistent with selection, or at least compare levels of genetic diversity at candidate loci to genome-wide levels to determine whether within population polymorphism is low relative to genomic background levels consistent with selective sweeps, for example.
As the revised version of the manuscript does not include the evaluation of selection pressures and genotype-environment association, the two comments above not further apply.

(4) It was not clear to me whether the raw FASTQ data have been made publicly available. The raw sequence data would be needed to evaluate how different filtering, mapping, and variant calling procedures affect downstream results (not just the VCF available from Dryad). Also, it would be helpful if the authors provided the data points from which summary statistics were calculated, e.g., the average depth of coverage for each sample (used to calculate the average per individual depth of coverage), heterozygosity for each individuals (used to estimate mean heterozygosity and perform statistical tests), etc. The authors have explained broadly how some quantities were calculated, e.g. heterozygosity was calculated with Stacks, however there is no mention of what inputs were used and where the exact code for carrying out analysis can be found. This is why I selected "No" for the question of whether the authors have made all data underlying the findings in their manuscript available.
We fully comply with the Data Availability policy and confirm that the raw data and the assembled GBS data (vcf file) of the Giant Toad Rhinella horribilis analyzed are available in Dryad (now clearly indicated in the revised submission). Importantly, we did not develop any specific code for the analyses performed; we followed the scripts provided within each of the programs used, while describing in detail all the parameters applied within the manuscript. Finally, the metadata (e.g. sampling locations, data per individual), the methods for all analyses, and the genetic results obtained are available in the main text and in the Supporting Information.

Minor Comments:
83–86: Change to "Identifying which environmental variables have higher leverage on patterns of functional connectivity is therefore essential to understanding whether local adaptations evolve in isolation or in the face of gene flow and what the main extrinsic cues shaping selective pressures are."
Done

184: Do the authors mean "NovaSeq lanes", not "lines"?
Yes! thanks for noticing; we rectified it

188: What do the authors mean by the Phred quality score of reads? That is, what aspect of read base, mapping, or alignment quality does the Phred quality of reads refer to?
We changed the wording for clarity to (lines 218-222): "The bioinformatic processing of the raw data comprised the following steps: demultiplexing raw reads with the function *process_radtags* in Stacks v.2.62 [39]; quality filtering with FastQC v.0.11.9 [40] using a Phred quality score <20 and removing adapters, as well as trimming reads to a minimum length of 100 bp with Trimmomatic v.0.36 [41]." The Phred quality score refers to the raw sequences for removing bases with low Phred quality scores (<20), as part of the pre-filters before calling SNPs.

195: Change "SNPs" to "SNP".
This line was deleted in the new version.

198: It's not clear what a minimum depth of 10 samples means. Are the authors alluding to a minimum number of samples with a minimum sequencing depth of coverage for retaining sites?
198– 99: Does maximum amount of missing data refer to a maximum number of individuals with missing genotypes?
199: What determined the minimum MAF cutoff of 10%? It would be helpful if the authors could justify why such a high MAF cutoff was used (and also what this means for inferring population structure when variation might be private to some subpopulations).
We are really sorry for the confusing writing of these methods. For your first comment, we were referring to the genotype depth, while for the second the filter excludes sites with missing genotypes in at least 20% of the total sample. Regarding the third, because we now worked with neutral datasets, we only applied mac filters.

In the revised version this entire section of the description of filtering protocols changed significantly. Lines 218-241 : "The total number of reads generated from sequencing was 1,187,625,595. The bioinformatic processing of the raw data comprised the following steps: demultiplexing raw reads with the function *process_radtags* in Stacks v.2.62 [39]; quality filtering with FastQC v.0.11.9 [40] using a Phred quality score <20 and removing adapters, as well as trimming reads to a minimum length of 100 bp with Trimmomatic v.0.36 [41]. We aligned the resulting reads to the *R. marina* reference genome using the function *BWA mem* in BWA v.0.7.1 [42]. Calling of single nucleotide polymorphisms (SNPs) was done with the *ref_map.pl* pipeline and the *populations* module in Stacks, using the parameters p=7, r=0.7.

From this first SNPs dataset and considering the objectives of our study, we built three datasets, one that included all the samples from the two landscapes (study-wide dataset) and two more by landscape (population-study-P1O and population-study-P2O). Additional quality filtering was done for each dataset with VCFtools v.0.1.13 [43], SNPfiltR v.1.0.2 [44] in R [45], and *populations* in Stacks. We provide a detailed description of the filtering processes and results in the Supplementary Methods. Briefly, we assessed allele balance using *filter_allele_balance* with the default parameters [46]; applied genotype depth and genotype quality using --minDP=5 and --minGQ=20, respectively [47]; only biallelic loci were retained; maximum allowed amount of missing data of 20% (--*max-missing*); and a string cut-off value of 10kb for the linkage disequilibrium (LD) filter (--*thin*) (see specific details in Supplementary Methods). We also removed potential paralogous sequences using a cut-off value of 2x mode, where "x" is per-SNP mean sequencing depth [48], and SNPs with observed heterozygosity >0.50 (--*max-obs-het*). Finally, we assessed a range of minor allele count (--*mac*) values (3 to 17; see Supplementary Methods for all the results), and chose a minor allele count of seven for both the study-wide and the population-study-P1O datasets and of five for the population-study-P2O."

We repeated all genetic diversity, structure and landscape analyses for this revised version of the manuscript.

We are confident that with all the bioinformatic and analytical changes and improvements made thanks to the reviewers' suggestions, the questions and predictions of our study were adequately evaluated. Moreover, results did not change while patterns became clearer.

I understand that cutoffs for bioinformatic filtering can be arbitrary, but these decisions can influence estimates of population genetic parameters and effect downstream inferences, and so I believe that it is generally good practice to justify chosen cutoffs. This ensures confidence in findings. I would suggest that the authors examine the genome-wide site frequency spectrum (SFS) for each population based on their filtered set of data. I suggest to examine the SFS because the proportion of SNPs belonging to each allele frequency category should be proportional to 1/x, where x is the allele frequency. While demography can skew the shape of the genome-wide SFS, it can generally only due so much, and excessive deviations from the expected distribution of allele frequencies can clearly point out if quality control provides a reliable set of SNPs, e.g., the SFS can inform at which point you accurately identify rare variants (so inform on MAF cutoffs), when SNP calling and allele frequency estimation is confounded by paralogy or other regions of the genome that are collapsed in a reference assembly or otherwise refractory to short read mapping. The reason why estimating the SFS accurately is so important is because many population genetic summary statistics on which the authors' analyses rely are direct functions of the SFS. For example, estimates of genetic diversity (Watterson's theta, nucleotide diversity, heterozygosity) are all direct functions of the SFS, while FST is a function of the joint SFS between multiple populations. Therefore, if there are clear problems in accurately estimating the SFS, which is fairly straight forward to identify given the known theoretical shape that it should approximately have, then these quantities will not be estimated accurately. Therefore, ensuring that the SFS for each subpopulation roughly aligns with the expected SFS will validate the bioinformatic processing and ensure that downstream results are reliable. If this is not the case then the authors will need to revise their quality control criteria. I recommend supplying plots of the SFS for each subpopulation and/or groups of samples with low structure (e.g. P10 and P20) calculated for sets of analyzed sites in the supplementary materials.

Also, the SFS (and hence quantities focused on by the authors) are sensitive to allelic dropout inherent to RADseq approaches, e.g. see https://doi.org/10.1111/mec.12276. It is not totally clear to me how the authors bioinformatic processing addressed this (I suspect it was how missing data was filtered, which also was not totally clear to me). This, again, is why I stress that examining the SFS for each subpopulation and/or group to ensure that it looks sane conditional on the set of quality controlled genomic sites would be a good idea.
As mentioned above, given the specific focus of this revised version, we did not use MAF.
Many thanks for this thorough comment, which is very helpful for the follow up work on the genomics and adaptation with our study system.

200–201: I think the authors mean that SNPs with a p-value less than 0.05 for a test of deviation from HWE were removed. As written, the authors would be removing sites with high evidence of being in HWE, which would be undesirable. I suspect the authors accidentally switched the inequality sign and mean "$p < 0.05$".
It was a typo error, we are sorry.
Based on the comments of another reviewer, we eliminated the HWE filter for the new datasets.

387–388: I suggest reporting standard deviation along with mean heterozygosity to provide better understanding of what the distribution of heterozygosity between P10 and P20 is.
Done

390–394: It seems that the authors have only reported results about genome-wide levels of differentiation within landscapes, i.e., within P10 and P20. I think it would be valuable to report how

diverged toads from the P10 landscape collectively are from P20 toads, that is, P10 vs P20 genome-wide FST, which will help genomically contextualize the comparisons of functional loci between P10 and P20 toads.
Done. We estimated the Fst value between landscapes and added the value in Results (0.0218) (lines 391-393).

Similarly, I suggest that the authors perform a joint PCA and admixture analyses of all toads (P10 and P20 pooled) to ensure that genome-wide patterns of genetic relationships are in line with expectations, which will (1) help validate the bioinformatic processing that yielded the set of SNPs upon which comparisons of adaptive loci rely, and (2) help with interpretation of reported differences (and similarities) in the genetic basis of environmental adaptation.
Yes indeed! As mentioned above, we built a study-wide (P1 and P2 together) dataset and performed structure analyses. Results (PCA and SNMF) clearly identified two distinct genetic groups (Fig 1d,e), namely P1O and P2O are genetically differentiated.

596–597: Where the authors state “low to medium density of temporary water bodies (natural and artificial)”, I suggest the authors reiterate what low–medium means with a range of exact values, for ease of interpreting these big takeaways.
We added this information, and now it reads: “Our findings showed an overall gradient where resistance was lower when water bodies were not very abundant (<0.5 in a scale from 0 to 1), with resistance rapidly increasing at higher values (>0.5). Low to medium density of temporary water bodies (natural and artificial), which are frequent in modified landscapes, allow for individual stepping-stone dispersal, facilitating genetic interchange, and likely sustaining the genetic diversity we observe in *R. horribilis* populations.”

637–639: “In fact, NOTCH was discovered in the frog Xenopus, in which it is active during early embryonic stages and in the development of the nervous system [100,101]”: It’s not clear to me why the focus on NOTCH in Xenopus cited here is relevant or supports why NOTCH-related processes confer adaptation to different environments. I would argue this statement is not needed given what follows.
The entire section was removed.
