# Supplementary material for: Functional connectivity patterns of the Giant Toad Rhinella horribilis in anthropogenically modified landscapes
Source: PLoS One. 2025 Oct 15;20(10):e0319111. doi: 10.1371/journal.pone.0319111 (PMC12527146; doi:10.1371/journal.pone.0319111)
Supplement: Attachment — Submitted filename: comments_to_authors_v2.pdf [file pone.0319111.s006.pdf]

I was Reviewer #1 for the previous submission. I am pleased to say that the authors have greatly improved the clarity of the manuscript and rigor of data filtering, analyses, etc. I also appreciate that the removal of the section concerning genomic signatures of local adaptation may not have been an easy decision. I want to clarify that the ideas that were presented in that section were interesting, and I hope to see a future paper focused on that topic. I have some remaining feedback, including two major concerns regarding 1) the omission of relevant landcover categories in the isolation by resistance modeling; and 2) the clarity of conclusions. Feedback related to these concerns and more minor comments are detailed in my line comments below.

- L28-29: This sentence is a bit challenging to read. What about "Anthropized environments often fragment native habitats and alter the movement of individuals across the modified landscape mosaic"? More broadly, many sentences in the abstract and throughout the paper have relatively complex structures that make them challenging to read (e.g., many interjecting phrases).
- L32: This sentence can be streamlined. E.g., "We used ddRAD-seq genomic data to study the genetic diversity, genetic structure, and functional connectivity of Giant Toad (Rhinella horribilis) populations across two landscapes with distinct levels of habitat modification."
- L30: The word "tolerance" here seems out of place since this study is focused on characterizing landscape features that affect population structure. The analyses do not directly address the extent to which the study species is tolerant to modified habitat. I think that this phrase can be omitted without changing the sentence much. E.g., "Deciphering the environmental factors associated with population genetic differentiation is essential for the conservation of wild populations inhabiting increasingly modified habitats."
- L36: "… associated with its functional connectivity…" Replace "its" with "the toad's" for clarity.
- L37: This sentence reads as though the differences in habitat modification are why toads in the two study areas are genetically differentiated. This is not clearly supported by the landscape genetics analyses, which primarily focused on water bodies, vegetation, and abiotic variables like solar radiation.
- L55-59: This sentence is quite long. I recommend splitting it up.
- L62: Replace "usually" with "often" or "can".
- L64: "and even thrive" seems a bit informal and is not adding much to the sentence.
- L64: Split the sentence before "facilitated." I.e., "Nevertheless, some species can persist~~, and even thrive~~, in modified areas. **Persistence can be** facilitated by

specific adaptations in their life history, ecology and functional traits that counteract the negative consequences of isolation."

- L66-67: This sentence again does not accurately describe the consequences of increased connectivity for local adaptation. Mutation is responsible for the evolution of genetic variants. Locally adaptive **genetic differentiation** (not locally adaptive variants) is what may not evolve when gene flow is strong enough to homogenize allele frequencies.
- L82: Replace "higher" with "high" since an explicit comparison is not being made.
- L81-84: The repeated discussion of local adaptation may not be warranted in the Introduction given that the tests for genomic signatures of spatially varying selection have been removed in this version of the manuscript. These sentences seem better suited for the Discussion section.
- L94: Remove the word "successful" and keep "common and abundant". I.e., "One of the most common and abundant amphibian species..."
- L98-99: Remove "(i.e., detrimental conditions)." It is not necessary given the earlier contents of the sentence.
- L99: Include the average weight, or a range of weights, when stating that the species has a medium-large body size. I.e., "Its medium-large body size (X kg), terrestrial habits..."
- L102-104: It is not clear to me how these ecological characteristics make this species particularly well-suited to the study question. Do these characteristics contrast with other amphibian species in the same general geographic area? And if so, is the authors' message here that studying the Giant Toad would thereby improve the representation of diverse ecology in research concerning anthropogenic impacts on amphibians?
- L104: Replace "anthropically" with "anthropogenically" here and throughout.
- L109-110: What life history traits led to the prediction that the two landscapes are likely genetically differentiated? Would the high dispersal capacity (L100) not make it likely that there is little genetic structure across the species' geographic range?
- L20-123: the claim that P1O is more strongly modified than P2O is still not clear to me. In Fig1, it seems that the landscapes might have similar amounts of human-modification but different spatial configurations of human-modified land. I.e., landcover categories in P2O appear more strongly spatially autocorrelated or "patchier". Perhaps the authors could provide a quantitative comparison of the environmental conditions in the two landscapes. E.g., what proportion of each landscape is in each landcover category?
- L195: Replace "string" with "stringent"?

- L205: It might be clearer to state the two levels of analysis as "at the total dataset level, considering all 15 localities together" and "within each of the P1O and P2O landscapes separately."
- L214 and throughout the manuscript: "*a priory*" should be replaced with "*a priori*".
- L232: The use of a t-test to evaluate differences in genetic summary statistics between landscapes seems inappropriate. Firstly, the authors do not state that they evaluated the normality and equal variance assumptions underlying the t-test. Secondly, the level of observation here may be the spatially distributed localities, which are not independent of one another. The authors should employ a more sophisticated analysis to test for differences between the landscapes. E.g., the authors could use the nlme package to fit a model with landscape as the focal predictor and specify the spatial autocorrelation structure with something like corGaus.
- L237-243: Please provide the pairwise correlation matrix for each landscape as a supplementary figure.
- L261-271: The manual assignment of cost/resistance values to rivers and roads in the MRDM analysis strikes me as odd because methods used later in the paper (ResistanceGA) can estimate these values objectively. ResistanceGA is capable of handling categorical data, so why not include roads and rivers in the set of models optimized with ResistanceGA? Isolation by barrier is essentially a specific sub-concept within the broader concept of isolation by resistance. Furthermore, the failure to account for roads and rivers in the ResistanceGA models could affect the inferred shapes of the relationships between other environmental predictors and landscape resistance, especially if certain values of other environmental predictors tend to co-occur with roads.
- L284-314: As stated previously, ResistanceGA can handle categorical predictors like landcover classes. Based on Fig.1, there are a few broad landcover categories present in both study areas that could affect connectivity among toad populations. It would be possible to explicitly test whether urban landcover and/or cropland are associated with high landscape resistance. Indeed, doing so would bolster the authors' claim that anthropogenic activity affects connectivity in this species.
- L294: Remove the phrase "all combinations of these parameters." Genetic algorithms like the one used in ResistanceGA are intended *not* to perform an exhaustive search of a large parameter space.
- L298: the phrase "maximize the relationship between pairwise genetic distances… and pairwise landscape distances" is not very clear. The authors should state which model fit statistic is being optimized by ResistanceGA's genetic algorithm. The default is log-likelihood (method = "LL" in the GA.prep function).

- L378: Table 2 appears out of order based on the presentation of the results in the main text. The current Table 2 should be relabeled as Table 1.
- L381-383: How do the authors conclude that the negative relationship between roads and FST in P2O means that roads do not impact connectivity? To me it would suggest that roads facilitate or enhance connectivity in P2O. Please clarify.
- L400: “distance” meaning geographic distance?
- L457-458: The authors state “Our findings show that the landscapes studied are genetically differentiated given their distinct degree of habitat modification.” I interpret this as the authors stating that habitat modification is responsible for the genetic differentiation observed between the two landscapes. It is not clear which results support this claim.
- L466: The authors state that “*R. horribilis* populations are not connected”, as indicated by genetic differentiation between the two landscape study areas. FST between the two landscapes is ~0.02 (L335), which does not support this strong claim.
- L469-470: The utility of SNP heterozygosity for determining whether a population has low or high genetic diversity in an absolute sense is not clear because these estimates are based only on loci *known to be polymorphic*. SNP heterozygosity is only useful in a relative sense when making comparisons within a given dataset (e.g., population A has higher diversity than population B).
- L496-510: The authors state that temporary water is important for connectivity among giant toad populations. However, the results show that higher values of kernel density probability of temporary streams are associated with higher landscape resistance in P1O (Fig5a,b). Similarly, very high values of temporary water bodies are associated with higher landscape resistance in P2O. The results appear to contradict the authors’ claim. Please clarify.
- L527-529: This section is titled “Conclusions and future directions,” but it only discusses local adaptation. Local adaptation is not addressed in this manuscript, so I suggest that this section’s title is changed to only “Future directions.”
- L535-537: The authors state “Also, including populations from areas with no anthropogenic impacts, to assess if their populations exhibit genetic variants that are fixed or specifically associated to these environments but absent from natural (conserved) ones.” I think the authors mean that they could do a test for spatially varying selection between natural and anthropogenically modified habitats, but this is not clear here.
- Table 2: The model labels (“road 1”, “road 2”…) are not very informative and heavily rely on additional information found in the supplement to be interpretable. I suggest the authors make this table more self-contained.

- Fig1a: The dark red points showing the two study areas are difficult to see due to the dark green background shading. Can the red be changed to a different color? E.g., yellow-filled points with black outlines?
- Fig1d: Please include the percent variance explained by each PC axis in the axis labels. This suggestion also applies to Fig2e and Fig3e.
- Fig1e: Are the individuals ordered in any way beyond the P1O vs P2O distinction? I am curious about where the P2O individuals with relatively high P1O ancestry were sampled. If not already done, it might be interesting to order the individuals by latitude. I suspect the aforementioned P2O individuals are located at the northern portion of P2O.
- Fig2b/c: Please re-code the colors to be more consistent between SNMF and TESS. Based on the ZAA and ZAB localities, the red cluster in SNMF seems to correspond closely to the green cluster in TESS. Comparison would be easier if red and green were switched for either panel b or c.
- Fig3b/c: Please flip the orientation of the colors to be more consistent between SNMF and TESS. I.e., blue appears on top in the SNMF plot but on the bottom in the TESS plot. Comparison would be easier if red was always on top or always on bottom in both panels.
- Supplementary Methods Table S1: It is not clear that the numbers in the table are SNPs. This should be indicated in the table caption and ideally within the table itself. Similarly, it is not clear that the numbers in parentheses in the "mac" column reflect different mac thresholds. I think transposing the table might make it clearer. I.e., have one column titled "Filter", which would contain the different filtering steps used as column names in the current table. Then add a second column titled "SNPs remaining", which would contain the numbers of SNPs at each filtering step. The same modifications should be made to Supplementary Methods Table S2.
- Figure S6: Please ensure all the panels use the same range of values for the x- and y-axes to enable comparisons between the landscapes. Please also include a panel showing isolation by distance across the total dataset (both P1O and P2O combined).
- Finally, I have a remaining concern regarding data availability. The authors state in their response "Importantly, we did not develop any specific code for the analyses performed; we followed the scripts provided within each of the programs used, while describing in detail all the parameters applied within the manuscript" as a justification for excluding scripts from the data repository associated with this manuscript. While the authors may not have developed custom software for their analyses, they must have written their own scripts (e.g., in R) to run the analyses they cited. It is important that repositories (e.g., Dryad or GitHub) contain the scripts

the authors wrote to perform analyses, as written descriptions of methods often end up being insufficient to exactly reproduce what was done. For example, the ResistanceGA analysis relies on the user writing one or more scripts with at least three different function calls and requires carefully formatted environmental and genetic data.
