# Supplementary material for: Functional connectivity patterns of the Giant Toad Rhinella horribilis in anthropogenically modified landscapes
Source: PLoS One. 2025 Oct 15;20(10):e0319111. doi: 10.1371/journal.pone.0319111 (PMC12527146; doi:10.1371/journal.pone.0319111)
Supplement: Attachment — Submitted filename: Response to reviewers_R2.pdf [file pone.0319111.s007.pdf]

Response to Academic Editor and reviewers (R2)

Comments from the Academic Editor

Thank you for submitting your manuscript to PLOS ONE. After careful consideration, we feel that it has merit but does not fully meet PLOS ONE's publication criteria as it currently stands. Therefore, we invite you to submit a revised version of the manuscript that addresses the points raised during the review process.

The manuscript has improved a great deal, with your revisions and the reviewers guidelines. The reviewer adds a few suggestions on wording and framing, and in some cases asks for clarifications or questions interpretation. Please fix these.

The main remaining issues are the following.

1. Take a closer look at the landscape genetics part, see comments by reviewer 1.
2. Provide copies of the scripts used for the analyses, like in R. This means you have to clean up the script and comment it, and ideally submit to Github with relevant datafile.

Response: We appreciate the enthusiastic evaluation and the new comments and suggestions. We carefully revised the landscape genetics part and made changes and improvements based on the reviewer's comments. Our point by point answers are included below; note that when line numbers are indicated, these refer to the clean version of the manuscript (without track changes).

We also now share the information and data as suggested: We compiled the scripts used in the study into five sections with the different analyses performed and include them in the Supplementary Methods file. We also include in a repository the raw sequence data and the vcf files for the study-wide and the two population-study datasets used. We indicate this for the revised version and include the FigShare doi: 10.6084/m9.figshare.29913284.

Line 54-Dont see how surviving human impact is key for long term evolution? Agree human impact is major and many organisms have to evolve to survive, but there I dont see need to talk about “long term evolution“?

Response: We rephrased as: “Adaptation to anthropized habitats is key for the persistence of species in such environments”

Line 81-This sentence is a bit convoluted, Here a suggestion “Identifying environmental variables that most strongly affect functional connectivity is therefore essential to understand the level of fragmentation, whether local adaptations evolve in isolation or in the face of gene flow and what may be the main selective pressures.”

Response: We thank for the suggestion and rephrased accordingly: “Identifying environmental variables that most strongly affect amphibian genetic patterns is therefore essential to understand their functional connectivity in modified landscapes.”

Line 105- and elsewhere

Why use the word “landscapes“ not just “areas“?

Response: From the first round of revision the reviewers suggested to use landscapes (instead of areas) as being more descriptive of the study objectives, with which we agreed.

Line 130-Add info on HOW many individuals were sampled.
Response: It is indicated in the Results (190 adults were sampled).

Line 144-Indicate that the PCA and admixture are estimated from XXX SNPs from ddRADseq.
Response: We added "based on 6034 SNPs".

Line 210-Seems to lack of info here. "We applied PCA to both the study wide and the population-study datasets, while DAPC only for the latter. We ran PCA with the glpca function in adegenet v.2.1.3 in R [50], retaining 50 and 26 factors for P1O and P2O, respectively."
PCA on whole set, how many factors were retained? And why keep different nr of factors for the two areas?
Response: We are sorry but we do not understand the question.
PCA on whole set? It is indicated that the PCA was applied to the study-wide dataset and the population-study datatset (lines 211-213; 218); what each of these datasets means is described in detail in a previous paragraph.
How many factors were retained? In line 220 it says "… retaining 50 and 26 factors for P1O and P2O, respectively."
Different number of factors were retained for each P1O and P2O based on what the analyses themselves indicated best.

Line 326-Maybe just skip "landscape" and refer to P10 and P20.
Response: Done

Comments from Reviewer 1
I was Reviewer #1 for the previous submission. I am pleased to say that the authors have greatly improved the clarity of the manuscript and rigor of data filtering, analyses, etc. I also appreciate that the removal of the section concerning genomic signatures of local adaptation may not have been an easy decision. I want to clarify that the ideas that were presented in that section were interesting, and I hope to see a future paper focused on that topic. I have some remaining feedback, including two major concerns regarding 1) the omission of relevant landcover categories in the isolation by resistance modeling; and 2) the clarity of conclusions. Feedback related to these concerns and more minor comments are detailed in my line comments below.
Response: We deeply appreciate and thank the reviewer for the enthusiastic comment; yes indeed, we are preparing a new manuscript with the local adaptation work.
In this revised version, we carefully revised and considered the main concerns (points 1) and 2)) and minor comments, which we explain in detail in our answers below.

• L28-29: This sentence is a bit challenging to read. What about "Anthropized environments often

fragment native habitats and alter the movement of individuals across the modified landscape mosaic"? More broadly, many sentences in the abstract and throughout the paper have relatively complex structures that make them challenging to read (e.g., many interjecting phrases).
Response: We thank for the suggestion and rephrased accordingly. We also revised throughout the manuscript and modified wording where we thought appropriate, along the abstract and other parts of the manuscript.

• L32: This sentence can be streamlined. E.g., "We used ddRAD-seq genomic data to study the genetic diversity, genetic structure, and functional connectivity of Giant Toad (Rhinella horribilis) populations across two landscapes with distinct levels of habitat modification."
Response: Done

• L30: The word "tolerance" here seems out of place since this study is focused on characterizing landscape features that affect population structure. The analyses do not directly address the extent to which the study species is tolerant to modified habitat. I think that this phrase can be omitted without changing the sentence much. E.g., "Deciphering the environmental factors associated with population genetic differentiation is essential for the conservation of wild populations inhabiting increasingly modified habitats."
Response: Done

• L36: "… associated with its functional connectivity…" Replace "its" with "the toad's" for clarity.
Response: Done

• L37: This sentence reads as though the differences in habitat modification are why toads in the two study areas are genetically differentiated. This is not clearly supported by the landscape genetics analyses, which primarily focused on water bodies, vegetation, and abiotic variables like solar radiation.
Response: We are sorry for the confusion. This line refers to genetic structure analysis conducted between the two landscapes (study-wide dataset). The results identified two distinct genetic groups (see lines 352-354; Fig 1d,e), namely that P1O and P2O are genetically differentiated.
We modified the text for clarity and now it reads: "Structure analysis between the two landscapes show that they are genetically differentiated given their distinct degree of habitat modification. Within landscapes, our results identified …"

• L55-59: This sentence is quite long. I recommend splitting it up.
Response: Thanks. We split them and added some text: "Pollution of soils and water bodies derived from agriculture, livestock and industrial activities are also markedly detrimental [6-8]."

• L62: Replace "usually" with "often" or "can".
Response: Changed to often

• L64: “and even thrive” seems a bit informal and is not adding much to the sentence.
Response: We deleted it.

• L64: Split the sentence before “facilitated.” I.e., “Nevertheless, some species can persist, and even thrive, in modified areas. Persistence can be facilitated by specific adaptations in their life history, ecology and functional traits that counteract the negative consequences of isolation.”
Response: Done

• L66-67: This sentence again does not accurately describe the consequences of increased connectivity for local adaptation. Mutation is responsible for the evolution of genetic variants. Locally adaptive genetic differentiation (not locally adaptive variants) is what may not evolve when gene flow is strong enough to homogenize allele frequencies.
Response: We deleted this sentence.

• L82: Replace “higher” with “high” since an explicit comparison is not being made.
Response: This line was modified as: “Identifying environmental variables that most strongly affect … ”

• L81-84: The repeated discussion of local adaptation may not be warranted in the Introduction given that the tests for genomic signatures of spatially varying selection have been removed in this version of the manuscript. These sentences seem better suited for the Discussion section.
Response: The reviewer is right! We modified this entire last sentence to: “Identifying environmental variables that most strongly affect amphibian genetic patterns is therefore essential to understand their functional connectivity in modified landscapes.”

• L94: Remove the word “successful” and keep “common and abundant”. I.e., “One of the most common and abundant amphibian species…”
Response: Done

• L98-99: Remove “(i.e., detrimental conditions).” It is not necessary given the earlier contents of the sentence.
Response: Done

• L99: Include the average weight, or a range of weights, when stating that the species has a medium-large body size. I.e., “Its medium-large body size (X kg), terrestrial habits…”
Response: We include it now (100 to 200 mm total length)

• L102-104: It is not clear to me how these ecological characteristics make this species particularly well-suited to the study question. Do these characteristics contrast with other amphibian species in the same general geographic area? And if so, is the authors’ message here that studying the Giant Toad would thereby improve the representation of diverse ecology in research concerning anthropogenic impacts on

amphibians?
Response: Yes, that is the case.

• L104: Replace "anthropically" with "anthropogenically" here and throughout.
Response: Done; it was the only case we used anthropically.

• L109-110: What life history traits led to the prediction that the two landscapes are likely genetically differentiated? Would the high dispersal capacity (L100) not make it likely that there is little genetic structure across the species' geographic range?
Response: We are sorry for the confusion. We predicted the two landscapes to be differentiated based on the fact that they have rather different levels of habitat modification and cover of land use classes. We modified the wording for clarity: "Based on the distinct landscapes studied and on *R. horribilis* life history traits, we respectively predicted (1) the two landscapes to be genetically differentiated; (2) low genetic diversity, high genetic structure and low gene flow (functional connectivity) among *R. horribilis* populations in the landscape with higher habitat modification; and (3) …"

• L120-123: the claim that P1O is more strongly modified than P2O is still not clear to me. In Fig1, it seems that the landscapes might have similar amounts of human-modification but different spatial configurations of human-modified land. I.e., landcover categories in P2O appear more strongly spatially autocorrelated or "patchier". Perhaps the authors could provide a quantitative comparison of the environmental conditions in the two landscapes. E.g., what proportion of each landscape is in each landcover category?
Response: For this revised version we calculated some quantitative measures that are included in a new table (Table S1) and in the description of the landscapes and land use classes (lines 120-125): "Each landscape has a different configuration, amount of vegetation cover, and land uses (Fig 1a-c, Table S1); Landscape 1 (P1O; high modification) has lower vegetation cover (47%), higher fragmentation (56.2; CONTAG index) and 50.64% modified land (pastures and technified agriculture, livestock and bare soil) in comparison with Landscape 2 (P2O; moderate modification) with 54.1% vegetation cover, fragmentation (50.6), and 43.5% modified land [33] (Table S1)."

• L195: Replace "string" with "stringent"?
Response: Done

• L205: It might be clearer to state the two levels of analysis as "at the total dataset level, considering all 15 localities together" and "within each of the P1O and P2O landscapes separately."
Response: We modified the description based on the suggestion: "Genetic structure analyses were conducted at two levels, one considering all 15 localities together (study-wide dataset) and the other within each of the P1O and P2O landscapes separately (population-study datasets)."

• L214 and throughout the manuscript: "a priory" should be replaced with "a priori".
Response: We apologize for this unintended mistake.

• L232: The use of a t-test to evaluate differences in genetic summary statistics between landscapes seems inappropriate. Firstly, the authors do not state that they evaluated the normality and equal variance assumptions underlying the t-test. Secondly, the level of observation here may be the spatially distributed localities, which are not independent of one another. The authors should employ a more sophisticated analysis to test for differences between the landscapes. E.g., the authors could use the nlme package to fit a model with landscape as the focal predictor and specify the spatial autocorrelation structure with something like corGaus.

Response: We eliminated the t-test and performed, as suggested, a linear model with the *gls* function with corGaus, considering spatial correlation and landscape as predictor for the three genetic metrics (Ho, He, Fis).

In the methods it now reads: "Statistical differences between landscapes were evaluated using a linear model, considering spatial correlation structure (*corGaus*), genetic diversity as the response variable, and landscape as the explanatory variable, using *gls* in nlme v.3.1 (https://CRAN.R-project.org/package=nlme). We also estimated the genetic differentiation between P1O and P2O with the pairwise fixation index (F_{ST}) with *pairwise.neifst* with hierfstat v.0.5-11 [56] in R."

And in the results: "Finally, genetic diversity (average observed and expected heterozygosity) was significantly (p<0.001) higher in P2O (*Ho*=0.139 ±0.0040, *He*=0.153 ±0.0049) than P1O (*Ho*=0.109 ±0.0022, *He*=0.118 ±0.0030), t-value=-18.211 and t-value=-15.706, respectively. In all sampling sites, the observed heterozygosity was lower than the expected heterozygosity, while low FIS values were inferred except for IPA in P1O, and SCB, TEB and PNC in P2O (Table S2), with not statistical differences between landscapes (t-value=-1.753, p=0.103)."

• L237-243: Please provide the pairwise correlation matrix for each landscape as a supplementary figure.

Response: We include a new table ("Table S3. Pearson correlation results of the environmental surfaces tested in the functional connectivity models. The correlation test was performed independently for each landscape. Correlated variables (>0.8) are in bold."), with the information as a matrix for each landscape.

• L261-271: The manual assignment of cost/resistance values to rivers and roads in the MRDM analysis strikes me as odd because methods used later in the paper (ResistanceGA) can estimate these values objectively. ResistanceGA is capable of handling categorical data, so why not include roads and rivers in the set of models optimized with ResistanceGA? Isolation by barrier is essentially a specific sub-concept within the broader concept of isolation by resistance. Furthermore, the failure to account for roads and rivers in the ResistanceGA models could affect the inferred shapes of the relationships between other environmental predictors and landscape resistance, especially if certain values of other environmental predictors tend to co-occur with roads.

Response: Thank you for your comment, and while what you mention is correct, we chose to model these variables to explicitly test the barrier isolation hypothesis. Because we explicitly wanted to test whether roads and rivers functioned as barriers, we classified these features as impermeable, which translates as the highest resistance values. With the MRDM tests, our intention was to capture a positive

or negative relationship between genetic distances and the distances associated with each barrier scenario.

• L284-314: As stated previously, ResistanceGA can handle categorical predictors like landcover classes. Based on Fig.1, there are a few broad landcover categories present in both study areas that could affect connectivity among toad populations. It would be possible to explicitly test whether urban landcover and/or cropland are associated with high landscape resistance. Indeed, doing so would bolster the authors' claim that anthropogenic activity affects connectivity in this species.
Response: Based on the suggestion we include a new categorical layer in the individual models that refers to land use (LU) in these landscapes. This is described in the Methods "We used infrared visualization to classify dense forest, secondary forest, bare soil, crops and pastures, urban areas and temporary water bodies, performing supervised classification by maximum likelihood in the satellite images (see [36]). Based on this classification we built a categorical land use surface (LU)." These results were added as well as a new Table S7"
Altogether, these land class surfaces we tested adequately describe anthropized landscapes.

• L294: Remove the phrase "all combinations of these parameters." Genetic algorithms like the one used in ResistanceGA are intended not to perform an exhaustive search of a large parameter space.
Response: We deleted all combinations

• L298: the phrase "maximize the relationship between pairwise genetic distances… and pairwise landscape distances" is not very clear. The authors should state which model fit statistic is being optimized by ResistanceGA's genetic algorithm. The default is log-likelihood (method = "LL" in the GA.prep function).
Response: We rephrased for clarity as: "The model fit statistic for the optimized resistance surfaces was the AICc (Akaike's information criterion corrected for small/finite sample size) [75], and a bootstrap resampling of the data to evaluate the robustness of the models."

• L378: Table 2 appears out of order based on the presentation of the results in the main text. The current Table 2 should be relabeled as Table 1.
Response: Table 1 is mentioned in the manuscript previously to the results section, as a reference of the best individual models.
Lines 327-330: "Finally, considering the best-supported individual models (see Table 1) and our connectivity hypotheses, we built four composite surfaces (aquatic, structural, biological-1 (facilitating) and biological-2 (limiting) models; see Table S5) to run a multivariate optimization approach."

• L381-383: How do the authors conclude that the negative relationship between roads and FST in P2O means that roads do not impact connectivity? To me it would suggest that roads facilitate or enhance connectivity in P2O. Please clarify.
Response: We thank the reviewer for pointing this out, which is true. We modified the text indicating that "these roads facilitate connectivity"

• L400: "distance" meaning geographic distance?
Response: yes, we added "geographic"

• L457-458: The authors state "Our findings show that the landscapes studied are genetically differentiated given their distinct degree of habitat modification." I interpret this as the authors stating that habitat modification is responsible for the genetic differentiation observed between the two landscapes. It is not clear which results support this claim.
Response: With the clarification and text changes we did in this revised version, which we explained above in other points mentioned by the reviewer, plus the results obtained, we think this is clear now:
In Introduction: "Based on the distinct landscapes studied and on *R. horribilis* life history traits, we respectively predicted (1) the two landscapes to be genetically differentiated; …"

In Methods: "Genetic structure analyses were conducted at two levels, one considering all 15 localities together (study-wide dataset) and the other within each of the P1O and P2O landscapes separately (population-study datasets)." …
"We applied PCA to both the study-wide and the population-study datasets, …"
"We tested SNMF from *K*=1 to *K*=3 with the study-wide dataset; …"

In Results: "Genetic structure analyses with the study-wide dataset clearly identified two distinct genetic groups, as shown by the PCA and the SNMF structuring results, which clearly separate P1O and P2O (Fig 1d,e); additionally, results indicated a F_{ST}=0.0218."

• L466: The authors state that "R. horribilis populations are not connected", as indicated by genetic differentiation between the two landscape study areas. FST between the two landscapes is ~0.02 (L335), which does not support this strong claim.
Response: We describe these results in a little more detail: "Genetic structure analyses with the study-wide dataset clearly identified two distinct genetic groups, as shown by the PCA and the SNMF structuring results, which clearly separate P1O and P2O (Fig 1d,e); additionally, results indicated a F_{ST}=0.0218."

• L469-470: The utility of SNP heterozygosity for determining whether a population has low or high genetic diversity in an absolute sense is not clear because these estimates are based only on loci known to be polymorphic. SNP heterozygosity is only useful in a relative sense when making comparisons within a given dataset (e.g., population A has higher diversity than population B).
Response: We here describe the overall genetic diversity (in a relative sense indeed) observed in the two *R. horribilis* populations as a whole, comparing the two landscapes studied (P1O and P2O).

• L496-510: The authors state that temporary water is important for connectivity among giant toad populations. However, the results show that higher values of kernel density probability of temporary streams are associated with higher landscape resistance in P1O (Fig5a,b). Similarly, very high values of

temporary water bodies are associated with higher landscape resistance in P2O. The results appear to contradict the authors' claim. Please clarify.

Response: The results indeed show that high spatial presence (concentration, density) of water bodies (both temporary and streams) limit functional connectivity (higher resistance).

Our discussion of these results in the lines indicated are concordant, in the terms that we describe "Our findings showed an overall gradient **where resistance was lower when water bodies were not very abundant (<0.5 in a scale from 0 to 1**), with resistance rapidly increasing at higher values (>0.5). Low to medium density of temporary water bodies (natural and artificial), which are frequent in modified landscapes, enable individual stepping-stone dispersal, facilitating genetic interchange and likely sustaining the genetic diversity we observe in *R. horribilis* populations. These results agree with previous studies showing that temporary streams associated with riparian vegetation and forested areas promote connectivity in urban and agricultural areas (e.g., [19,87,88,93-95])."

• L527-529: This section is titled "Conclusions and future directions," but it only discusses local adaptation. Local adaptation is not addressed in this manuscript, so I suggest that this section's title is changed to only "Future directions."

Response: Thanks for the suggestion. We deleted Conclusions and made some changes to the text (lines 551-556)

• L535-537: The authors state "Also, including populations from areas with no anthropogenic impacts, to assess if their populations exhibit genetic variants that are fixed or specifically associated to these environments but absent from natural (conserved) ones." I think the authors mean that they could do a test for spatially varying selection between natural and anthropogenically modified habitats, but this is not clear here.

Response: It is true, the wording was confusing. We modified the text for clarity as: "It would be interesting to expand our study with *R. horribilis*, to determine if it presents signals of parallel evolution in landscapes representing different degrees of human modification. Also, comparing such gradient with populations from areas with no anthropogenic impacts to assess if their populations exhibit genetic variants that are fixed or specifically associated to natural (conserved) landscapes."

• Table 2: The model labels ("road 1", "road 2"…) are not very informative and heavily rely on additional information found in the supplement to be interpretable. I suggest the authors make this table more self-contained.

Response: We agree with the reviewer and to facilitate interpretation of Table 2, we decided to include figure S2 in the main text (new Figure 2).

• Fig1a: The dark red points showing the two study areas are difficult to see due to the dark green background shading. Can the red be changed to a different color? E.g., yellow-filled points with black outlines?

Response: We changed the colors as suggested. It really looks better now.

• Fig1d: Please include the percent variance explained by each PC axis in the axis labels. This suggestion also applies to Fig2e and Fig3e.
Response: As suggested, we include the variation explained per PC axis in the corresponding figures.

• Fig1e: Are the individuals ordered in any way beyond the P1O vs P2O distinction? I am curious about where the P2O individuals with relatively high P1O ancestry were sampled. If not already done, it might be interesting to order the individuals by latitude. I suspect the aforementioned P2O individuals are located at the northern portion of P2O.
Response: Thanks for the suggestion. We modified the image, and now the samples are sorted by latitude (which should be noted, also correlates with altitude) from left to right for each landscape. Interestingly, P2O individuals with higher P1O ancestry are located in the southern portion of P2O.

• Fig2b/c: Please re-code the colors to be more consistent between SNMF and TESS. Based on the ZAA and ZAB localities, the red cluster in SNMF seems to correspond closely to the green cluster in TESS. Comparison would be easier if red and green were switched for either panel b or c.
Response: Many thanks for the suggestion, we modified the figure, matching the blue, green and red colors (genetic clusters) in the SNMF and TESS graphs.

• Fig3b/c: Please flip the orientation of the colors to be more consistent between SNMF and TESS. I.e., blue appears on top in the SNMF plot but on the bottom in the TESS plot. Comparison would be easier if red was always on top or always on bottom in both panels.
Response: Great suggestion again, many thanks. We flipped the ancestry bars orientation.

• Supplementary Methods Table S1: It is not clear that the numbers in the table are SNPs. This should be indicated in the table caption and ideally within the table itself. Similarly, it is not clear that the numbers in parentheses in the “mac” column reflect different mac thresholds. I think transposing the table might make it clearer. I.e., have one column titled “Filter”, which would contain the different filtering steps used as column names in the current table. Then add a second column titled “SNPs remaining”, which would contain the numbers of SNPs at each filtering step. The same modifications should be made to Supplementary Methods Table S2.
Response: We attempted to transpose the tables, but it became more confusing. We therefore modified the description of the table, which we believe is clearer now. “*Rhinella horribilis* study-wide post-filtering process. The number of retained SNPs after each filter are shown in each column, in a step-by-step order. Column names are allele balance (0.25 - 0.75 allele frequency); minDP: genotype depth; minGQ: genotype quality; only biallelic loci; max-missing: 20% of missing data allowed; thin: linkage disequilibrium 10kb; max-meanDP: site-mean-depth mode*2; max het: maximum observed heterozygosity allowed; mac: minor allele count. The mac column shows the different mac values tested in parenthesis. In bold, the final dataset used for the study-wide analyses.” Same applies for table 2 in supplementary methods (we modified it accordingly).

• Figure S6: Please ensure all the panels use the same range of values for the x- and y-axes to enable comparisons between the landscapes. Please also include a panel showing isolation by distance across the total dataset (both P1O and P2O combined).

Response: We cannot apply this suggestion because each landscape has a different size; thus the x-axis range is from 0 to 3.0 (log geographic distance values) in P1O and form 0 to 2.5 in P2O. Extending this in P2O to 3 will only show a blank area in the right side of the graph.

Also, each genetic distance has a different range of values based on how they are estimated. We modified the graphs for the y-axis, which now have the same range of values when depicting the same genetic distance, allowing to compare both landscapes for Fst and for Dps, respectively.

Finally, we consider that a panel of isolation by distance across the total data set is not appropriate, based on the results that significantly separate the two landscapes and because, in accordance, the main focus of the study was analyzing the genetic patterns per landscape.

• Finally, I have a remaining concern regarding data availability. The authors state in their response "Importantly, we did not develop any specific code for the analyses performed; we followed the scripts provided within each of the programs used, while describing in detail all the parameters applied within the manuscript" as a justification for excluding scripts from the data repository associated with this manuscript. While the authors may not have developed custom software for their analyses, they must have written their own scripts (e.g., in R) to run the analyses they cited. It is important that repositories (e.g., Dryad or GitHub) contain the scripts the authors wrote to perform analyses, as written descriptions of methods often end up being insufficient to exactly reproduce what was done. For example, the ResistanceGA analysis relies on the user writing one or more scripts with at least three different function calls and requires carefully formatted environmental and genetic data.

Response: We compiled the scripts used in the study into five sections with the different analyses performed and include them in the Supplementary Methods file. We also include in a repository the raw sequence data and the vcf files for the study-wide and the two population-study datasets used. We indicate this in the rebised version and include the FigShare doi: 10.6084/m9.figshare.29913284
