# Supplementary material for: Functional connectivity patterns of the Giant Toad Rhinella horribilis in anthropogenically modified landscapes
Source: PLoS One. 2025 Oct 15;20(10):e0319111. doi: 10.1371/journal.pone.0319111 (PMC12527146; doi:10.1371/journal.pone.0319111)
Supplement: Attachment — Submitted filename: Response to reviewers.docx [file pone.0319111.s008.docx]

27 September 2025

**Response to reviewers**

The one comment of the reviewer was that “All good, except the data sharing is imperfect. The figshare link is to another study/data”.

We are sorry for the confusion, the FigShare link was correct, but was not available yet (it was in building process) because we were working out on how to share the huge amount of information we have from our GBS sequencing.

We finally chose to upload the data to the NCBI (BioProject PRJNA1335177) and leave in the FigShare repository the rest of the shared information (https://doi.org/10.6084/m9.figshare.29913284). Both links are now active and published (you can check them directly).

We have clarified this in the corresponding section in the submission system as: “The demultiplexed Illumina reads of the Giant Toad *Rhinella horribilis* were uploaded to NCBI SRA database under accession number PRJNA1335177. The final assembled and filtered datasets (vcf files) used for the study-wide and the two population-study analyses are shared in the FigShare repository, https://doi.org/10.6084/m9.figshare.29913284. Sampling locations, data per individual, methods for all analyses, and genetic results are available in the main text and in the Supporting Information. The R scripts used in the study were compiled into five sections with the different analyses performed, which are included in the Supplementary Methods pdf file.”
